# Irradiation induces DJ-1 secretion from esophageal squamous cell carcinoma cells to accelerate metastasis of bystander cells via a TGF-β1 positive feedback loop

**DOI:** 10.1186/s13046-022-02471-6

**Published:** 2022-08-26

**Authors:** Junjie Gu, Yuanyuan Sun, Jiahang Song, Ruiling Zhao, Xiaoke Di, Yumeng Zhang, Xiaolin Ge, Shu Zhang, Yun Gu, Xinchen Sun

**Affiliations:** 1 The First School of Clinical Medicine, Nanjing Medical University, Nanjing, China; 2 Department of Radiation Oncology, The First Affiliated Hospital of Nanjing Medical University, No.300 Guangzhou Road, Nanjing, 210029 China; 3 Department of Endocrinology, The First Affiliated Hospital of Nanjing Medical University, Nanjing, China; 4 Core Facility Center, The First Affiliated Hospital of Nanjing Medical University, Nanjing, China; 5 Department of Thoracic Surgery, Lian Shui People’s Hospital, Huai’an, 223400 China

**Keywords:** DJ-1, TGF-β1, HSC70, Smad3, TSP1, Esophageal squamous cell carcinoma, Radiation-induced bystander effect, Tumor metastasis

## Abstract

**Background:**

Radiation-induced bystander effect (RIBE) can promote tumor metastasis contributing to the failure of radiotherapy for esophageal squamous cell carcinoma (ESCC). Aberrant expression of DJ-1 has been identified in ESCC; however, the relationship between DJ-1 and RIBE in ESCC remains unknown.

**Methods:**

We detected DJ-1 in the serum and cell supernatants by enzyme-linked immunosorbent assay (ELISA) and evaluated tumor metastasis by phenotypic experiments in vivo and in vitro. RNA-seq, mass spectrometry, western blot (WB), immunoprecipitation (IP), and dual-luciferase reporter assays were performed to explore the underlying mechanisms.

**Results:**

DJ-1 was highly expressed in the serum of patients with ESCC receiving radiotherapy and was significantly overexpressed in the medium of ESCC cells receiving irradiation. DJ-1 promoted tumor metastasis via the TGF-β1 pathway. Mechanistic studies revealed that DJ-1 bound to HSC70 to promote Smad3 phosphorylation and nuclear aggregation in a protein-interaction manner, which activated the transcription of Thrombospondin-1 (TSP1). Subsequently, the activation of TGF-β1 by TSP1 re-promoted Smad3 phosphorylation and nuclear aggregation, constituting a positive feedback loop to strengthen the metastasis of ESCC cells, which was effectively blocked by LY2109761 and LSKL. Moreover, higher levels of serum DJ-1 in patients with ESCC were related to a poorer prognosis of radiotherapy.

**Conclusions:**

Irradiation can induce ESCC cells secreting DJ-1. Secreted DJ-1 enters bystander cells to initiate activation of the TGF-β1 pathway via the DJ-1/HSC70/Smad3 signaling axis. The TSP1/TGF-β1/Smad3 positive feedback pathway constitutes the core pathway that promotes ESCC metastasis. DJ-1 is a useful biomarker for predicting the efficacy of radiotherapy and a potential therapeutic target for reversing RIBE in ESCC.

**Graphical Abstract:**

Schematic diagram showing the underlying mechanism that irradiation-induced secretion of DJ-1 accelerates the metastasis of bystander ESCC cells.

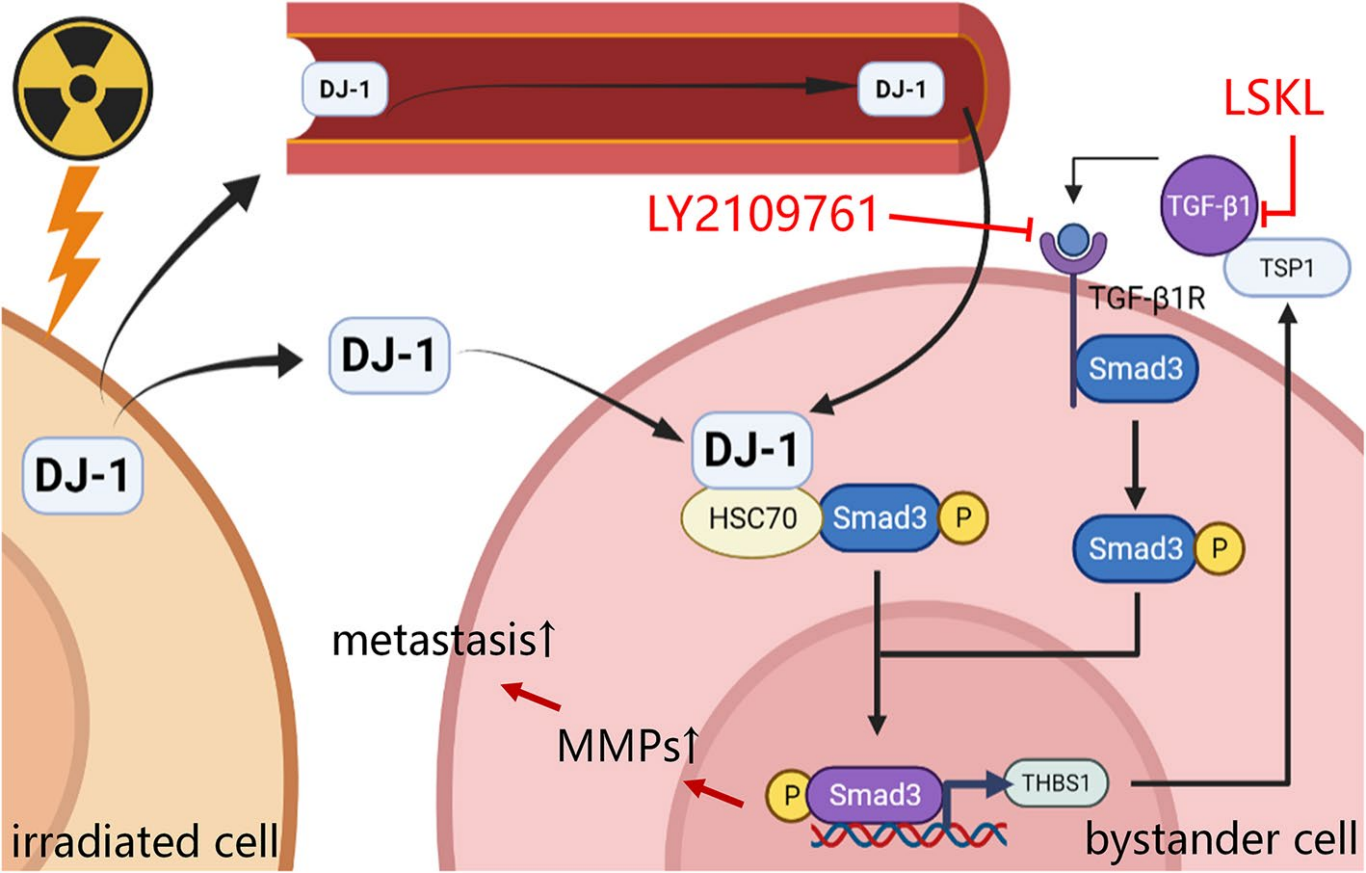

**Supplementary Information:**

The online version contains supplementary material available at 10.1186/s13046-022-02471-6.

## Background

Esophageal cancer is one of the most invasive tumors, ranking eighth and sixth in global morbidity and mortality, respectively. More than 70% of patients with esophageal cancer have esophageal squamous cell carcinoma (ESCC), with particularly high incidence rates in eastern Asia and several regions of Africa [1]. Owing to the difficulty of early diagnosis, the vast majority of patients with ESCC are diagnosed at an advanced stage. Even after surgery, radiotherapy, and chemotherapy, most patients experience recurrence and metastasis, and the 5-year survival rate varies between 10 and 33% [2]. The most fundamental reason for treatment failure is the metastasis; however, the mechanism of metastasis has not been fully clarified. Therefore, revealing the mechanism of metastasis of ESCC is key to improving the survival rate of patients with ESCC.

Radiation-induced bystander effect (RIBE) is one of the reasons for radiotherapy failure. RIBE refers to the phenomenon of biological changes in non-irradiated cells due to the signal transmission of irradiated cells. Experimental evidence has linked RIBE to the activation of invasion and metastasis in breast cancer, lung adenocarcinoma, and squamous cell carcinoma [3–5], highlighting the potential role of RIBE in reducing tumor metastasis and improving the efficacy of radiotherapy.

DJ-1/PARK7 is a highly conserved homodimer protein that was initially cloned as a putative oncogene capable of transforming NIH3T3 cells in cooperation with H-Ras [6]. Many studies have shown that DJ-1 is overexpressed in prostate cancer, pancreatic cancer, colon cancer, etc., and is positively correlated with tumor metastasis and negatively correlated with patient survival [7–9]. In ESCC, patients with high nuclear expression of DJ-1 have a higher rate of distant metastasis within one year after surgery [10]. Moreover, DJ‑1 promotes cell proliferation and tumor metastasis in esophageal squamous cell carcinoma via the Wnt/β‑catenin signaling pathway [11]. Although these studies suggest that the expression of DJ-1 is associated with the distant metastatic potential of ESCC, the relationship between DJ-1 and irradiation and whether it causes RIBE has not yet been reported.

In this study, we found that irradiation could induce ESCC cells to secrete DJ-1, and that the secreted DJ-1 promoted the metastasis of bystander non-irradiated cells, which was related to the positive feedback activation of the TGF-β1 pathway. As a result, we speculate that DJ-1 may be a potential biomarker for evaluating the prognosis of radiotherapy and a synchronous therapeutic target to enhance the efficacy of radiotherapy in ESCC.

## Methods

### The Cancer Genome Atlas (TCGA) and Gene Expression Omnibus (GEO) database analyses

We extracted data on 169 esophageal adenocarcinoma and squamous cell carcinoma cases from TCGA database (https://portal.gdc.cancer.gov/) to analyze differential expression, correlation, and patient survival. The data of GSE53625 were obtained from the GEO database (https://www.ncbi.nlm.nih.gov/gds/) and analyzed by differential expression, correlation and survival analyses.

### Patients and specimens

Between April 2018 and May 2021, 177 ESCC blood samples were obtained from the Department of Radiation Oncology at the First Affiliated Hospital of Nanjing Medical University. Table S1 shows the clinical characteristics of 177 patients. Of the 177 patients,46 patients received a similar therapeutic regimen, and their blood samples were collected again after receiving cumulative 40 Gy doses of radiotherapy. Table S2 lists the clinical characteristics and follow-up information for the 46 patients. Following 30 min incubation at 37 °C after blood collection, the samples were centrifuged at 3000 rpm for 10 min. We then extracted the supernatant to obtain serum samples. A total of 36 tumor tissue samples and nearby normal esophageal tissue samples were obtained from patients with ESCC who underwent tumor resection. All tissue samples were collected from the Lianshui County People’s Hospital. Table S3 lists the clinical characteristics of the 36 patients. Fresh tumor and non-tumor tissues were frozen in liquid nitrogen to preserve their integrity. Clinical staging was based on the seventh edition of the AJCC on Cancer Staging Manual. All procedures were in line with the ethical standards of the Human Trials Commission (institutional and national) and the Helsinki Declaration of 1975, as revised in 2000. All the patients provided informed consent to participate in the study. The study protocol was approved by the Ethics Committee and Institutional Review Board of the First Affiliated Hospital of Nanjing Medical University.

### Enzyme-linked immunosorbent assay (ELISA)

Cytokine concentrations were determined using a human DJ-1 ELISA kit (Invitrogen, USA) according to the manufacturer’s instructions. Briefly, serum or cell supernatant samples were added to the culture plate, followed by the addition of antibodies, streptavidin-HRP and TMB. The absorbance of each well was measured at 450 nm using a Pro-11 Multiskan FC spectrophotometer (Thermo, USA).

### Cell culture, irradiation methods and co-culture model construction

Human ESCC cell lines (KYSE-30, KYSE-150, KYSE-450, ECA-109, TE-1 and TE-10) were obtained from the American Type Culture Collection (ATCC, USA). The cells were cultured in RPMI1640 medium (Gibco, USA) containing 10% fetal bovine serum (Gibco, USA) at 37 °C in a humidified atmosphere with 5% CO_2_. The cells in the IR group were irradiated with 2, 4, 6, 8, or 10 Gy ionizing X-rays from a medical linear accelerator (Precise Accelerator, Elekta, Sweden). The irradiation field was confined within the culture dish or disk, and the dosing rate was 1.439 Gy/min. The direct co-culture model was constructed using Transwell inserts (8 μm, Corning, Germany). Firstly, 2 × 10^5^ irradiated cells were seeded in a 24-well plate. After irradiation, the transwell inserts were placed into each well of the culture plate, and then 5 × 10^4^ unirradiated cells were seeded in the inserts. In the indirect co-culture model, unirradiated cells were cultured with the medium of the irradiated cells. Figure S1 shows the direct and indirect co-culture models.

### Western blot

ESCC cells were lysed with RIPA buffer (KeyGEN, China). The total protein lysate (40 μg per lane) was loaded onto 6–12.5% Bis–Tris polyacrylamide mini gels (Epizyme, China). SDS-PAGE was run at 120 V for 1.5 to 2 h. Proteins were transferred to PVDF membranes (Roche, Switzerland) by wet transfer for 60–90 min at 100 V. Membranes were blocked in 5% nonfat dry milk in Tris-buffered saline supplemented with Tween 20 (0.1%) (TBS-T) or phosphate-buffered saline (PBS) for 60 min at room temperature. After blocking, membranes were cut horizontally to examine multiple proteins of different sizes on each gel. Membranes were incubated on a plate shaker overnight at 4 °C with primary antibodies diluted in TBS-T. Membranes were extensively washed with TBS-T (minimum 3 × for 10 min), followed by incubation with appropriate horseradish peroxidase-conjugated secondary antibodies diluted in TBS-T with 5% non-fat dry milk for 60 min at RT on a plate shaker. The membranes were extensively washed with TBS-T (minimum 4 × for 15 min). Protein bands were visualized using enhanced chemiluminescence (ECL) method. Multiple film (Tanon, China) exposures, ranging from 2 s to 2 min, were performed for optimal image analysis. The antibodies used are listed in Table S4. Glyceraldehyde-3-phosphate dehydrogenase (GAPDH) was used as an endogenous control to normalize the protein loading.

### Recombinant protein, small interfering RNA (siRNA), plasmid and inhibitor

Human PARK7/DJ1 protein (His-tag) was obtained from Sino Biological (Beijing, China). Active recombinant human TGF-β1 was purchased from Abcam (Cambridge, USA). The two proteins were stored at -80 °C until use. siRNAs targeting HSPA8, Smad3, and THBS1 were purchased from RiboBio (Guangzhou, China). The Smad3 plasmid vector and THBS1 wild-type or mutant plasmids were obtained from GeneCopoeia (MD, USA). LY2109761 and LSKL were purchased from MedChemExpress (NJ, USA). The negative vector, si-NC, and PBS were used as the control groups. ESCC cell lines were seeded in 6-well or 24-well plates 24 h prior to siRNA, plasmid, or inhibitor transfection at 70–80% confluence, and then mixed with jetPRIME transfection reagent (Polyplus, CA) according to the manufacturer’s instructions. All the sequences are listed in Table S5.

### Cell migration and invasion experiments

Either 2 × 10^5^ ESCC cells or DJ-1 (800 pg) were placed into each well in 1 ml of 10% RPMI1640 in a 24-well plate. After 8 Gy ionizing radiation X-ray for ESCC cells, transwell inserts were placed into each well containing 5 × 10^4^ unirradiated cells in 500 μl of appropriate culture media. Then, TGF-β1 (5 ng/ml), LSKL (17.46 μM), or LY2109761 (20 μM) were added to the culture medium, and the cells in the inserts were transfected with siRNA in advance if needed. Plates were cultured under normal conditions for 24 h, after which the inserts were removed, fixed in 4% paraformaldehyde (Biosharp, China) for 2 h, and then stained with 0.1% crystal violet (Beyotime, China) for 2 h. For the invasion assay, transwell inserts were pre-treated with 80 mL of Matrigel (Corning, Germany), and the remaining steps were the same as those used for the migration assay. Finally, the cells were visualized and photographed under a microscope (Nikon, Japan), and the number of cells per field was calculated.

The wound-healing assay was performed using an indirect co-culture model. ESCC cells were seeded in 6-well plates and allowed to grow to 100% confluence. A scratch was made using a P200 pipette tip, and the migration distance was measured 0 and 24 h after scratching.

A 3D tumor spheroid invasion assay was conducted using an indirect co-culture model to better simulate the invasion environment of tumor cells. A total of 200 µL of cell suspension containing approximately 10,000 cells was added into a 96-well U-shaped bottom microporous plate treated with Nunclon Sphera in advance (Thermo Scientific, USA). After incubation for 96 h, the cellular aggregates were collected and seeded into 100 µL of Matrigel. Following polymerization at 37 °C, the gel was overlaid with 200 µL of RPMI1640 with 10% FBS for 72 h. The motion of the cells was observed under a fluorescence microscope (Thunder DMi8, Leica, Germany).

### Animal experiments

Animal experiments were approved by the Institutional Animal Care and Use Committee of Nanjing Medical University (IACUC-2104040). After random assignment and anesthetization, nude mice were injected with 2 × 10^6^ cells suspended in 200 µL of PBS into the tail vein (*n* = 5 per group). The next day, the nude mice were injected with recombinant protein DJ-1(14.67ug) [12] through the tail vein. Meanwhile, the nude mice of the corresponding groups were injected intraperitoneally with LSKL (1 mg/kg) [13] or LY2109761 (50 mg/kg) [14] if needed. An IVIS imaging system (Caliper Life Sciences, USA) was used to monitor metastatic progression 10 min after intraperitoneal injection (150 mg/kg) of d-luciferin and potassium salt D (Yeasen, China) dissolved in DMSO. Luciferase signal intensity was maintained at the same scale. Mice were sacrificed after 6 weeks, and the lungs were removed for hematoxylin–eosin (H&E) staining. All operations were in line with the laboratory animal management norms.

### Immunofluorescence assay

In total, 5 × 10^4^ cells were seeded into a confocal laser cuvette. After 24 h of cell climbing, 4% paraformaldehyde was fixed for 1 h, 0.25% Triton X-100 was ruptured for 1 h, and 5% bovine serum albumin was blocked for 1 h. Cells were immunostained with antibodies overnight at 4 °C, washed, and incubated with fluorescently labeled secondary antibodies (Abcam, MA, USA) at 37 °C for 1 h, and nuclei were stained with 6-diamidino-2-phenylindole (DAPI) (Beyotime, China). A confocal laser scanning microscope (Leica, Germany) was used for observation and imaging.

### Co-immunoprecipitation (CoIP)

Cells were collected in cold PBS and lysed with RIPA buffer (50 mM Tris (pH 7.4), 150 mM NaCl, 1 mM EDTA, 0.1% SDS, 1% Nonidet P-40 (NP-40), 0.5% sodium deoxycholate, 0.5 mM DTT, and protease inhibitor). The lysates were diluted 2- to fourfold in dilution buffer (50 mM Tris (pH 7.4), 100 mM NaCl, 1 mM EDTA, 0.1% NP-40, 10% glycerol, and protease inhibitor). Then, 2–5 μg of antibodies were added to the diluted cell lysates, and the mixtures were incubated overnight at 4 °C. The following day, the protein complexes were isolated using magnetic Protein-A Magnetic Beads (CST, USA) for 2 h at 4 °C with rotation. The bead-antibody-protein complexes were then washed four times with wash buffer (50 mM Tris (pH 7.4), 125 mM NaCl, 1 mM EDTA, and 0.1% NP-40) and boiled for western blot analysis.

### RNA-seq and bioinformatics analysis

Sequencing was performed using 2 × 100 cycles (paired-end reads, 100 nucleotides) for all samples on an Illumina NovaSeq6000 instrument. Reads were quantified using salmon v0.14.1 (genome GRCh38), and differential analysis was performed using the R package DESeq2 (R version 4.0.3, DESeq2 version 1.30.1). No statistical methods were used to determine sample size. RNA-seq experiments were performed in triplicate. To identify as many differential genes as possible in the model’s dataset, genes that harbored a differential expression associated with logFC higher than 0.7 and FDR lower than 0.05. Kyoto Encyclopedia of Genes and Genomes (KEGG) analyses of differentially expressed genes (DEGs) were also performed according to the gene annotations provided by the DAVID online tool (v6.8). Pathway significance was expressed as a *P*-value calculated by right-tailed Fisher’s exact test, which indicates the possibility that the correlation between DEGs from our dataset and a given process/function is due to random chance.

### Liquid chromatography combined with tandem mass spectrometry (LC–MS/MS)

Mass spectrometry assays were performed according to the manufacturer’s instructions with minor modifications. The total protein was extracted and digested overnight with protease. The digested peptide mixture was dried and treated with 0.1% trifluoroacetic acid (TFA) (Sigma-Aldrich, USA). After diluting the 5μL samples, we used an LTQ Orbitrap Velos Pro mass spectrometer (Thermo Scientific, Germany) coupled with an Ultimate 3000 RSLC Nano System (Dionex, USA) to recover proteins and perform proteomic analysis of total proteins, which were identified using Proteome Discoverer 1.4 software (Thermo Fisher Scientific, USA), and the resulting original file was imported into the UniProt KB/Swiss-Prot database for searching. For the database search, the mass tolerances of the precursor and fragmentation were set at 10 ppm and 0.8 Da, respectively. Peptides with a false discovery rate of < 1% (q-value < 0.01) and proteins with an area value lower than 1 × 10^6^ were discarded. Proteins that met the following criteria were considered differentially expressed: ≥ 2 peptides and ≥ 95% confidence, and an average fold change in protein levels ≥ 2.00 or ≤ 0.50. (Student’s t-test, *p* < 0.05).

### Luciferase reporter assay

cDNAs with firefly luciferase, which contained the full-length sequence of THBS1 were cloned into pGL4.23-control vectors (Promega, USA). For the mutant reporter plasmids, 10 bases were replaced with opposite bases in the THBS1 motif. The plasmid sequences are listed in Table S5. Pre-treated ESCC cells were seeded into 6-well plates followed by co-transfection with 0.5 μg of wild-type or mutated THBS1 reporter plasmids and 25 ng pRL-TK plasmids (Renilla luciferase reporter vector) using the jetPRIME Polyplus kit. After 24-36 h, cells were harvested, and luciferase activity was assessed using the Dual-Glo Luciferase system (Promega, USA). This was normalized to the pRL-TK activity. Each experiment was conducted in triplicate.

### Tumor immunohistochemistry (IHC) and immunofluorescence (IF)

Tumor tissues were fixed in 4% paraformaldehyde in PBS for 24 h, embedded in paraffin blocks, and sectioned (5 μm). Slides were then stained with anti-rabbit primary antibodies. Signals were developed using the Vectastain Elite ABC Universal Plus kit, peroxidase (Vector Laboratories, Cat: PK-8200), or fluorescently labelled secondary antibodies. Cell nuclei were labeled with hematoxylin or DAPI. An Olympus IX81 fluorescence microscope was used to photograph representative peroxidase staining or fluorescence samples. The antibodies used for the immunohistochemistry and immunofluorescence assays are listed in Table S4.

### Quantitative real-time PCR

Total RNA was extracted from cells or tissues using TRIzol reagent (Invitrogen, USA), according to the manufacturer’s instructions. cDNA synthesis was performed using a PrimeScript RT Reagent Kit (TaKaRa, Japan). RT-PCR reactions were performed on a StepOnePlus Real-Time PCR System (Applied Biosystems, MA, USA) using a SYBR qPCR Master Mix kit (High ROX Premixed) (Vazyme, China) according to the manufacturer’s instructions. The expression of the target genes was normalized to that of GAPDH. The primers used for amplification are listed in Table S5.

### Statistical analysis

All experiments in this study were performed in triplicate. Differences between groups were determined using the non-parametric Kruskal–Wallis test or Mann–Whitney U test with Bonferroni correction. The patients were divided into high and low-expression groups based on the median gene expression level. Kaplan–Meier analysis was used to compare prognosis in patients with ESCC. STATA 14.0, SPSS 22.0 and GraphPad Prism 7.0 software were used to perform statistical analysis, and a *p* value < 0.05 was considered statistically significant (*p < 0.05, **p < 0.01, ***p < 0.001, ****p < 0.0001).

## Results

### DJ-1 is upregulated in ESCC samples and positively correlated to tumor progression

**Fig. 1:**
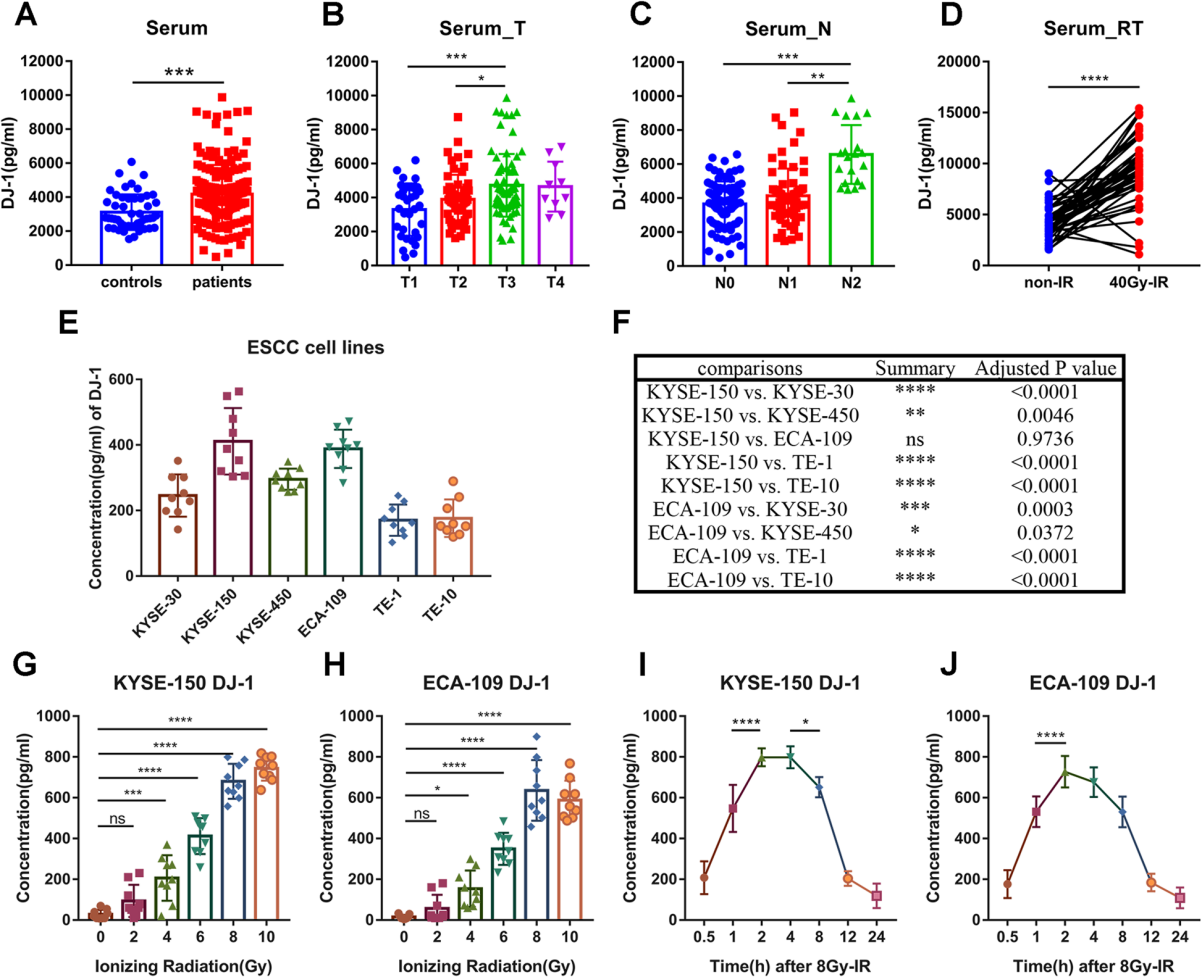
Serum DJ-1 expression correlates tumor progression and upregulates from ESCC cells for irradiation. **A** ELISA results of DJ-1 levels in serum of ESCC patients (*n* = 177) and normal donors (*n* = 50). **B-C** The expression of DJ-1 in serum of ESCC patients at multiple stages (T and N) by ELISA. **D** ELISA results of DJ-1 levels in serum of ESCC patients (*n* = 46) at the point of before and after radiotherapy (40 Gy). **E–F** ELISA results of DJ-1 expression in medium of 6 ESCC cell lines at the point of 2 h after 6 Gy-irradiation. **G-H** ELISA results of DJ-1 expression in medium of K150 and E109 cells at the point of 2 h after receiving multiple doses (0, 2, 4, 6, 8 and 10 Gy) of irradiation. **I-J** ELISA results of DJ-1 expression in medium of K150 and E109 cells at multiple timing (0.5, 1, 2, 4, 8, 12 and 24 h) after receiving 8 Gy irradiation. *p < 0.05, **p < 0.01, ***p < 0.001, ****p < 0.0001

The TCGA database was used to analyze the mRNA expression profiles of DJ-1/PARK7 in normal esophageal tissues (*n* = 10), ESCC tissues (*n* = 81), and EA tissues (*n* = 78), and it was found that the expression of DJ-1/PARK7 was significantly upregulated in ESCC tissues (Fig. S2A). In addition, the mRNA levels of DJ-1/PARK7 in patients with T3 stage ESCC were higher than those in patients with T1 stage ESCC (Fig. S2B). Consistent with the TCGA data analysis results, GEO data (GSE53625) analysis also showed that DJ-1/PARK7 was highly expressed in ESCC tumor tissues (Fig. S2C). Kaplan–Meier survival analysis indicated that DJ-1/PARK7 expression was negatively correlated with survival in patients with ESCC (Fig. S2D, *n* = 179, *p* = 0.0084). Furthermore, the expression of DJ-1 was detected in the serum of 177 ESCC patients and 50 normal donors and verified the conclusion of its high expression (Fig. 1A). Moreover, the expression of DJ-1 in serum with T3 stage was higher than that in T1 and T2 stage (1.43 times and 1.21 times) (Fig. 1B), and DJ-1 in serum with N2 stage was also higher than that in N0 and N1 stage (1.80 times and 1.59 times) (Fig. 1C). These results suggested that DJ-1 expression is positively correlated with ESCC development.

### Irradiation induces secretion of DJ-1 from ESCC cells

In 46 pairs of serum samples from ESCC patients before and after cumulative 40 Gy dose radiotherapy, the expression of DJ-1 in the serum of patients after radiotherapy was much higher than that before radiotherapy (Fig. 1D). To explore whether the elevated DJ-1 in serum was secreted by ESCC cells, we examined the DJ-1 levels by ELISA in the cell supernatants of six ESCC cell lines (KYSE-30, KYSE-150, KYSE-450, ECA-109, TE-1 and TE-10) after irradiation. The results suggested that, at the point of 2 h after 6 Gy-irradiation, DJ-1 was detected in the supernatants of all 6 cell lines and the two cell lines with the highest DJ-1 content in the cell supernatant were KYSE-150 and ECA-109 (Fig. 1E and F). We then selected these two cell lines for detailed content-dose-time analysis. These two ESCC cell lines (KYSE-150 and ECA-109) were irradiated with 0, 2, 4, 6, 8, and 10 Gy doses, and DJ-1 levels in the medium were measured at 0.5, 1, 2, 4, 8, 12, and 24 h after irradiation. The results indicated that the DJ-1 content in the medium reached a peak at 2 h after 8 Gy irradiation, which was approximately 798 and 727 pg/ml (Fig. S3A and B). At 2 h, with the increase in radiation dose, DJ-1 levels in the medium gradually increased, and plateaued after the dose increased to 8–10 Gy (Fig. 1G and H). The dose-time gradient after 8 Gy irradiation showed that the level of DJ-1 in the medium increased within 0–2 h, maintained a certain level at 2–4 h, and decreased rapidly to the initial level after 8 h (Fig. 1I and J). Meanwhile, Western blotting was used to detect the expression levels of DJ-1 in cells receiving 8 Gy irradiation at the corresponding time points. We found that the relative levels of DJ-1 reached their minimum at 2 h after irradiation (8 Gy) in both KYSE-150 and ECA-109 cell lines (Fig. S3C-F). These results demonstrated that irradiation induces the transport of DJ-1 from the intracellular to the extracellular regions of ESCC cells.

### Exogenous DJ-1 enhances the metastasis of ESCC cells in vitro and vivo

**Fig. 2:**
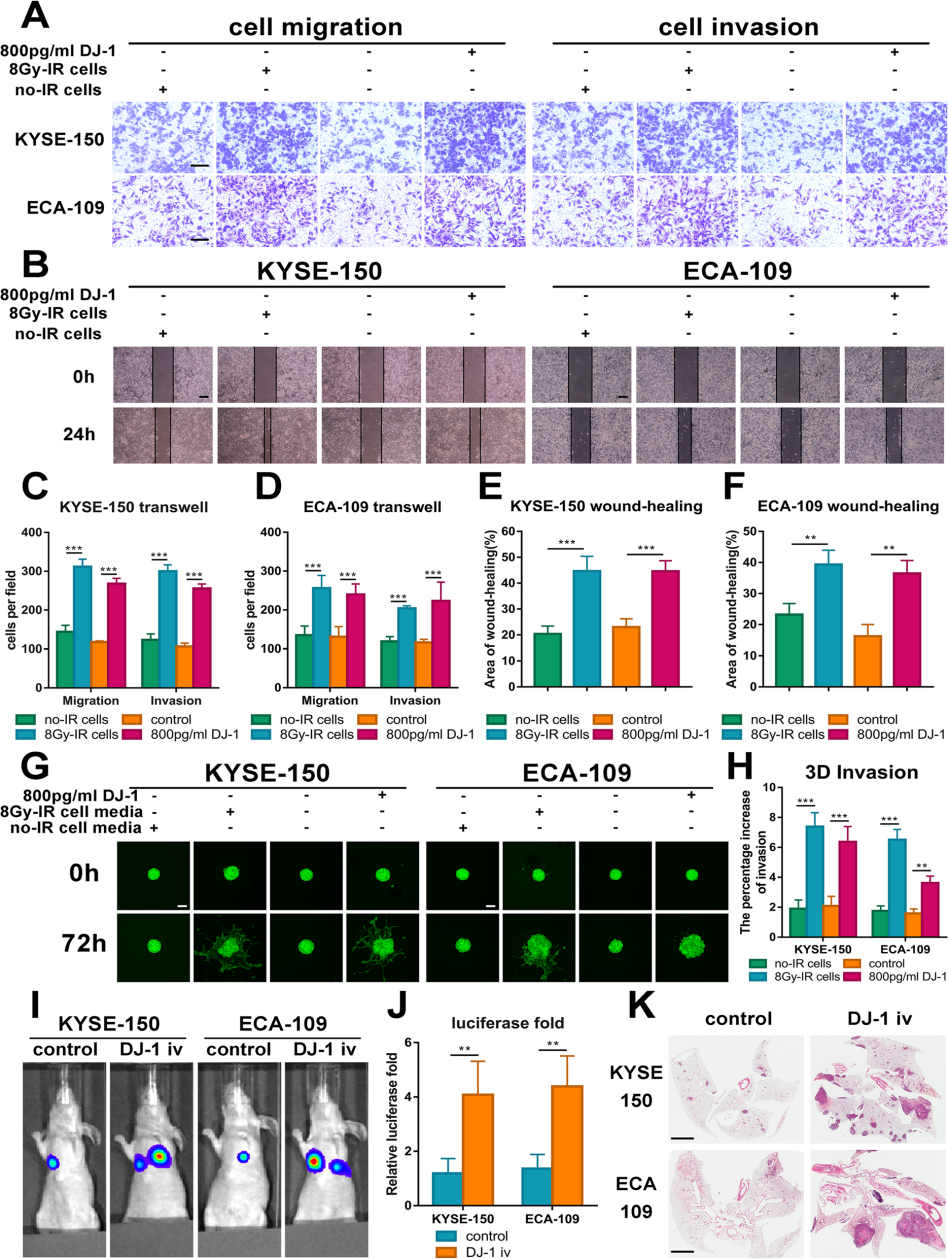
Exogenous DJ-1 enhances the metastasis of ESCC cells in vitro and vivo. **A** Representative images of transwell assays for bystander ESCC cells co-culture with irradiated cells or exogenous DJ-1. Scale bars, 100 μm. **B** Representative images of wound-healing assays for bystander ESCC cells co-culture with irradiated cells or exogenous DJ-1. Scale bars, 200 μm. **C-D** The statistical graphs of transwell assays presented in part A. **E–F** The statistical graphs of Wound-healing assays presented in part B. **G-H** Representative IF images and the statistical graph of the 3D tumor spheroid invasion assay for bystander cells (green) co-culture with irradiated cells or exogenous DJ-1. Scale bars, 200 μm. **I-J** Representative photographs of metastatic nodules were taken from nude mice injected DJ-1 protein by an IVIS imaging system. Quantification of the luciferase was shown. **K** H&E staining was used to characterize the lung metastatic nodules. Scale bar, 2 mm. **p < 0.01, ***p < 0.001

Since DJ-1 is an important signal transduction molecule, we speculated that DJ-1 secreted by ESCC cells may be associated with RIBE. First, in vitro experiments were performed. To simulate the occurrence process of RIBE, ESCC cells irradiated with 8 Gy were co-cultured with unirradiated bystander cells for 24 h. Transwell and wound healing assays demonstrated that co-culture with irradiated ESCC cells enhanced the migration and invasion of bystander K150 and E109 cells (Fig. 2A-F). Interestingly, when we used exogenous DJ-1 to stimulate cells, the migration and invasion of bystander cells were also strengthened (Fig. 2A-F). In addition, we obtained the same result through a 3D tumor spheroid invasion assay (Fig. 2G and H). Next, to generate a pulmonary metastasis model, K150 and E109 cells stably transfected with luciferase were injected into the tail vein of nude mice to determine the effect of DJ-1 on ESCC tumor metastasis. As expected, exogenous DJ-1 through tail vein injection greatly promoted the invasion of ESCC cells and aggravated pulmonary metastasis compared to the control group (Fig. 2I-K). Altogether, in vitro and in vivo experiments confirmed that exogenous DJ-1 reinforces the metastasis of ESCC cells.

### Exogenous DJ-1 promotes metastasis of bystander ESCC cells by activating TGF-β1 pathway

**Fig. 3:**
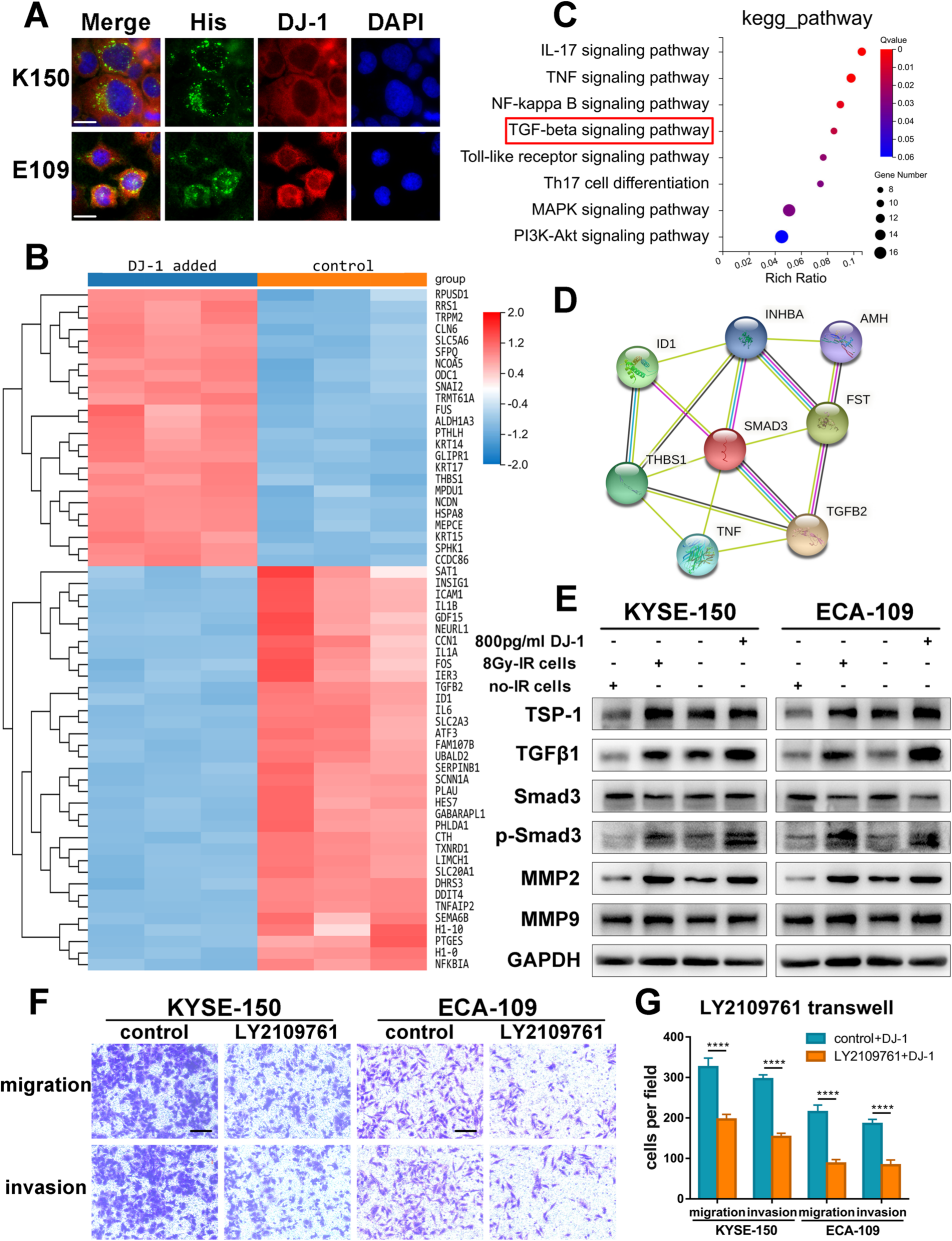
Exogenous DJ-1 promotes metastasis of bystander ESCC cells by activating TGF-β1 pathway. **A** Representative IF images of the intracellular localization of exogenous DJ-1 (red) tagged by His-tag (green) in ESCC cells detected using immunofluorescence microscopy. Scale bar, 20 μm. **B** Heatmaps of 59 representative DEGs screened from RNAseq analysis. **C** KEGG pathway enrichment analyses showed top activated gene sets ordered by rich ratio and the TGF-β1 signaling pathway was enriched after DJ-1 stimulation. **D** A network of proteins enriched in the TGF-β1 signaling pathway and encoded by DEGs from RNAseq results. **E** Western blot results of prominent proteins correlated with the TGF-β1 signaling pathway in bystander ESCC cells co-cultured with irradiated cells or DJ-1. **F-G** Representative images and the statistical graph of transwell assays for bystander ESCC cells measured by LY2109761. Scale bars, 100 μm. ****p < 0.0001

To explore the mechanism by which exogenous DJ-1 promotes metastasis, we first determined the localization of DJ-1 in ESCC cells. We labeled the recombinant protein DJ-1 with a His-tag and added it to the culture medium. Using immunofluorescence staining, we found that the His-tag DJ-1 was highly localized in the cytoplasm and partly in the nucleus (Fig. 3A). Fluorescence co-localization analysis showed that the site of His and DJ-1 double staining could be regarded as a recombinant DJ-1 protein, which strongly proved that DJ-1 can be freely transferred between ESCC cells (Fig. S4A).

Having determined the intracellular localization of DJ-1, we inferred that DJ-1 might play a role in signal transmission. To gain insight into the molecular mechanism of exogenous DJ-1 action on bystander cells, we analyzed the transcriptomes of bystander cells with and without exogenous DJ-1. The whole results of RNA-seq are shown in Table S6. According to the screening criteria of logFC > 0.7 and FDR < 0.05, 434 differentially expressed genes (DEGs) were discovered (Figs. 3B and S4B). KEGG enrichment was performed to demonstrate the top overrepresented canonical pathways sorted by *p*-value annotation for DEGs. The results indicated that the TGF-β1 pathway related to tumor metastasis was activated, which could be the reason for the metastasis of bystander ESCC cells (Fig. 3C). Based on the prediction of the protein interaction network of DEGs enriched in the TGF-β1 pathway, we found that Smad3 was the core of the network (Fig. 3D).

To verify the results of RNA-seq analysis, western blot was conducted to detect the expression of TGF-β1 pathway proteins and its downstream factors. The results showed that the ratio of p-Smad3/Smad3 increased, and the expression of TSP1, TGF-β1, and MMPs proteins increased to varying degrees (Fig. 3E). To investigate whether the TGF-β1 signaling pathway participates in DJ-1-induced metastasis, an inhibitor of the TGF-β1 signaling pathway (LY2109761) was used. As shown in Fig. 3F and G, LY2109761 reduced DJ-1-induced metastasis of bystander ESCC cells. Similar results were obtained in the 3D tumor spheroid invasion assay (Fig. S4C and D).

### Exogenous DJ-1 induces metastasis by binding to HSC70 in ESCC cells

**Fig. 4:**
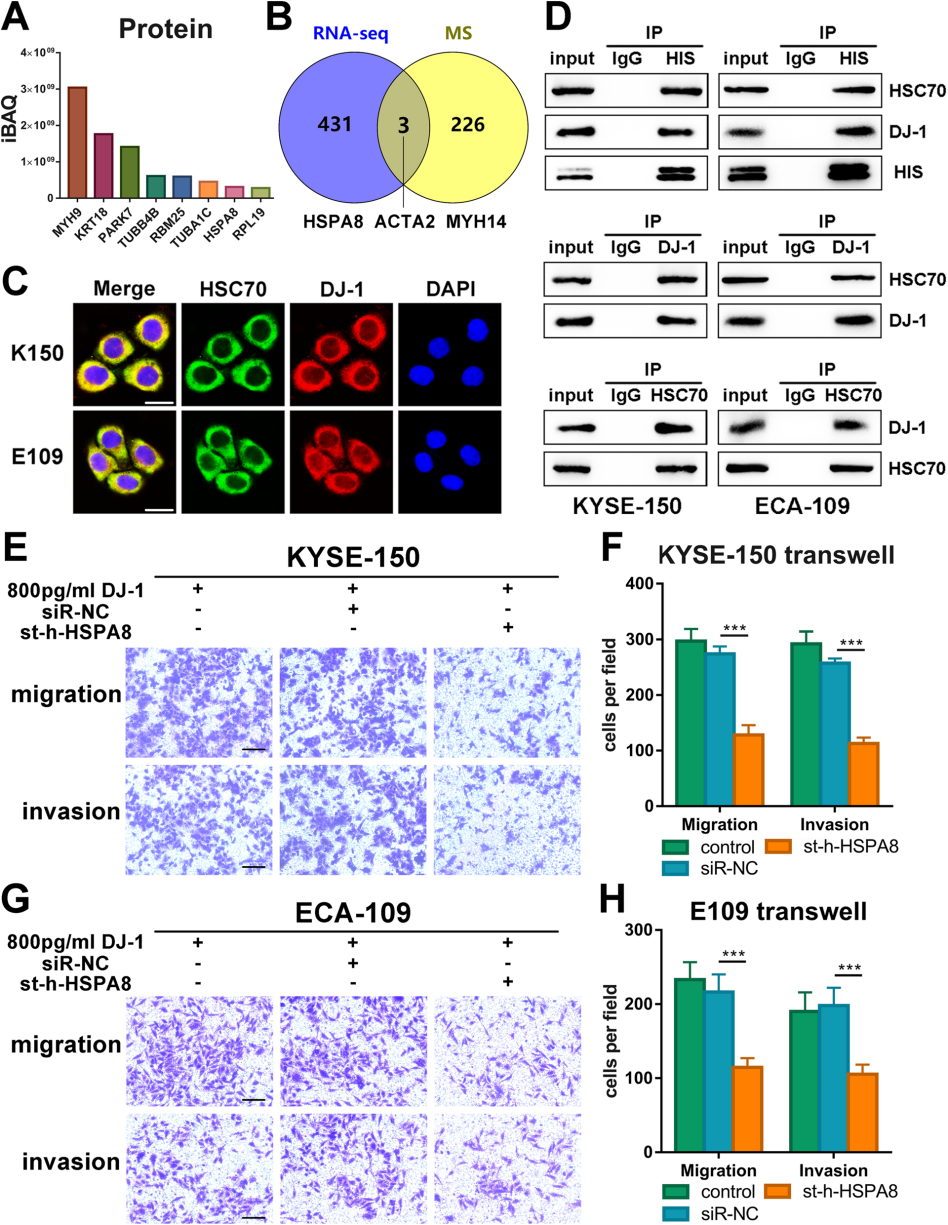
Exogenous DJ-1 promotes metastasis by binding to HSC70 in ESCC cells. **A** Results of proteins identified from LC–MS/MS ordered by the score of iBAQ. **B** The targets of intersection between proteins identified from LC–MS/MS and DEGs screened from RNAseq. **C** Representative IF images of colocalization of DJ-1 (red) and HSC70 (green) in K150 and E109 cells detected using immunofluorescence microscopy. Scale bar, 20 μm. **D** Coimmunoprecipitation analysis demonstrated that exogenous DJ-1 could interact with HSC70. **E–H** Representative images and the statistical graph of transwell assays for bystander K150 and E109 cells transfected with siRNA targeting HSC70. Scale bars, 100 μm. ***p < 0.001

To further determine the regulatory mechanism of DJ-1 in the TGF-β1 pathway, we performed liquid chromatography-tandem mass spectrometry (LC–MS/MS) in K150 cell samples co-immunoprecipitated with the DJ-1 antibody. Figure S5A shows the co-immunoprecipitation samples stained with Coomassie Brilliant Blue after electrophoresis. A total of 2392 proteins were identified according to the iBAQ values (Fig. 4A and Table S7). To increase the accuracy of identification of interacting proteins by MS, we combined the protein data with the transcriptome data from RNA-seq for a comprehensive analysis. Three proteins (HSPA8, ACTA2 and MYH14) were identified by overlapping the identified proteins and RNA-seq DEGs (Fig. 4B).

Previous studies have reported that HSPA8/HSC70 promotes TGF-β-induced Smad2/3 activation in fibroblast NRK-49F cells [15]. Additionally, it has been shown that the upregulation of HSPA8/HSC70 is associated with poor prognosis in patients with multiple cancers and stimulates tumor immune responses or drug resistance [16–19]. We inferred that HSC70 may be an effective protein in DJ-1 regulating the TGF-β1 pathway and promoted metastasis of ESCC cells. To verify the interaction between DJ-1 and HSC70, we found that DJ-1 and HSC70 were co-localized in the cytoplasm of K150 and E109 cells using immunofluorescence (Fig. 4C, Fig. S5C and D). Co-immunoprecipitation and western blotting further demonstrated a protein interaction between DJ-1 and HSC70 (Fig. 4D).

To further investigate the role of HSC70 in DJ-1-induced metastasis, we transfected siRNA targeting HSPA8/HSC70 into K150 and E109 cells. The transfection efficiency was verified by western blotting (Fig. S5B). Surprisingly, HSPA8/HSC70 siRNA transfection drastically decreased the migration and invasion of K150 and E109 cells induced by exogenous DJ-1 in transwell and 3D tumor spheroid invasion assays (Fig. 4E-F and Fig. S5E-F). These results indicated that exogenous DJ-1 promotes metastasis by binding to HSC70 in ESCC cells.

### Exogenous DJ-1 acting on HSC70 contributes to Smad3 phosphorylation and nuclear aggregation in ESCC cells

**Fig. 5:**
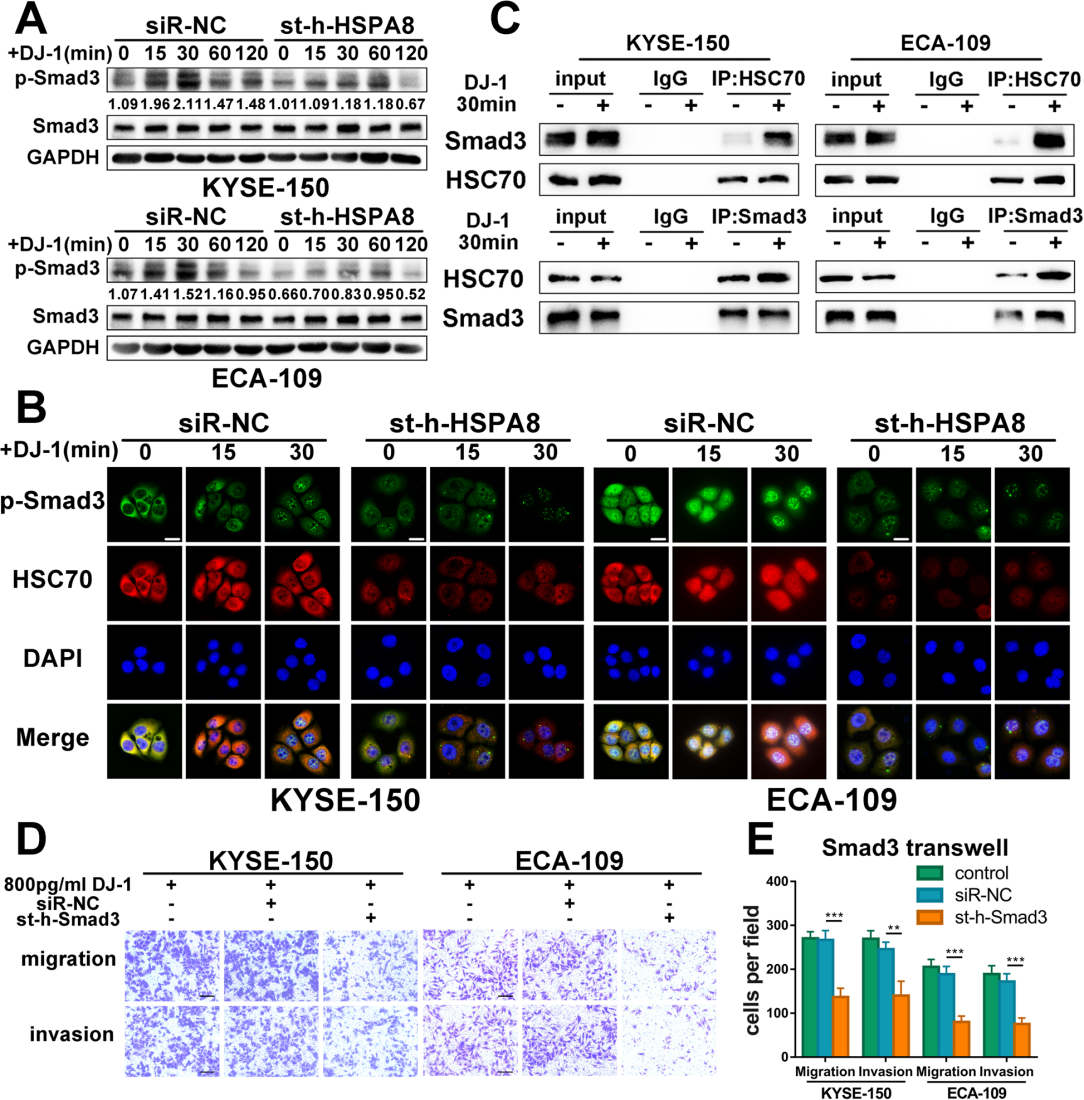
Exogenous DJ-1 acting on HSC70 promotes Smad3 phosphorylation and nuclear aggregation in ESCC cells. **A** Western blot results of Smad3 phosphorylation status relative to total Smad3 in K150 and E109 cells transfected with siRNA targeting HSC70 at the timing (0, 15, 30, 60, 120 and 240 min) after DJ-1 stimulation. **B** Representative IF images of p-Smad3 (green) and HSC70 (red) expression in K150 and E109 cells transfected with siRNA targeting HSC70 at the timing (0, 15 and 30 min) after DJ-1 stimulation. Scale bar, 20 μm. **C** Coimmunoprecipitation analysis demonstrated that HSC70 could interact with Smad3 in attendance of DJ-1. **D-E** Representative images and the statistical graph of transwell assays for bystander K150 and E109 cells transfected with siRNA targeting Smad3. Scale bars, 100 μm. **p < 0.01, ***p < 0.001, ****p < 0.0001

After identifying HSC70 as a downstream target of DJ-1, we speculated that the DJ-1/HSC70 complex may be concerned in the activation of Smad3. First, we transfected HSC70 siRNA or control siRNA into K150 and E109 cells. After stimulation with exogenous DJ-1, the phosphorylation status of Smad3 (p-Smad3) was examined by western blotting from 0 to 120 min. The ratio of p-Smad3/Smad3 reached a peak at the time point of 30 min in the control group, while the upward trend disappeared in cells transfected with HSC70 siRNA (Fig. 5A).

Next, the effect of HSC70 on the nuclear translocation of p-Smad3 was examined by immunofluorescence microscopy. As shown in Fig. 5B, both K150 and E109, in cells transfected with control siRNA, nuclear localization of p-Smad3 (green) was increased by DJ-1 stimulation. In contrast, in cells transfected with HSC70 siRNA, the level of HSC70 was completely suppressed (red), and the nuclear accumulation of p-Smad3 was also decreased (green) compared with the control group. The fluorescence intensity was significantly different between the two groups (Fig. S6A and B).

To explore the involvement of HSC70 in DJ-1-induced Smad3 signaling, the interaction between HSC70 and Smad3 was examined in K150 and E109 cells. The cells were treated for 30 min with or without exogenous DJ-1 and cell lysates were prepared with lysis buffer. HSC70 and Smad3 were immunoprecipitated from the lysate using specific antibodies. The immunoprecipitate was examined by western blotting using antibodies against HSC70 and Smad3. As shown in Fig. 5C, a small amount of Smad3 was immunoprecipitated with anti-HSC70 antibody in untreated cells. Upon stimulation with DJ-1, the levels of Smad3 increased in cells. Meanwhile, a low level of HSC70 was immunoprecipitated with an anti-Smad3 antibody in untreated cells. HSC70 expression also increased in cells stimulated by exogenous DJ-1. Collectively, these results suggested that the interaction between HSC70 and Smad3 is enhanced upon stimulation with exogenous DJ-1.

We also examined the effects of Smad3 on DJ-1-induced metastasis. We used siRNA to knock down the expression of Smad3 in ESCC cells and verified the knockdown efficiency by western blotting (Fig. S6C). As expected, knockdown expression of Smad3 significantly reduced DJ-1-induced migration and invasion of K150 and E109 cells in transwell and 3D tumor spheroid invasion assays (Fig. 5D-E and Fig. S6D-E). In summary, the above results demonstrated that DJ-1 binding to HSC70 accelerates the phosphorylation and nuclear aggregation of Smad3, which enhances the metastasis of ESCC cells.

### Exogenous TGF-β1 reactivates Smad3 without the presence of HSC70

**Fig. 6:**
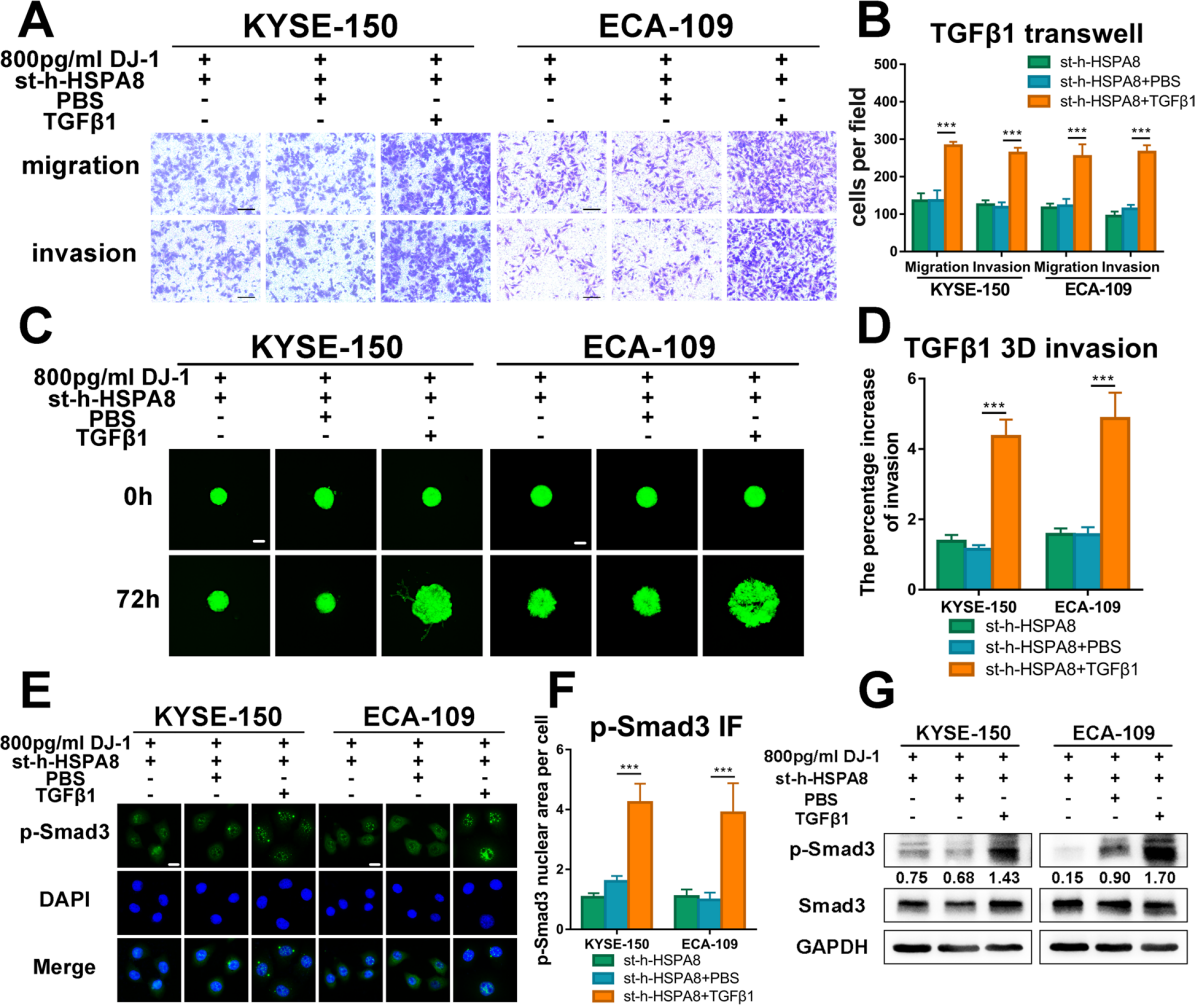
Exogenous TGF-β1 reactivates Smad3 without the presence of HSC70. **A-B** Representative images and the statistical graph of transwell assays for bystander HSC70-knockdown K150 and E109 cells stimulated by TGF-β1. Scale bars, 100 μm. **C-D** Representative IF images and the statistical graph of 3D tumor spheroid invasion assays for bystander HSC70-knockdown K150 and E109 cells (green) stimulated by TGF-β1. Scale bars, 200 μm. **E–F** Representative IF images of p-Smad3 (green) expression in HSC70-knockdown ESCC cells stimulated by TGF-β1 at the timing (240 min) after DJ-1 stimulation. Scale bar, 20 μm. G. Western blot results of Smad3 phosphorylation status relative to total Smad3 in HSC70-knockdown ESCC cells stimulated by TGF-β1. ***p < 0.001

TGF-β1 is a secretory growth factor that leads to the activation of Smad or non-Smad pathways [20, 21]. Ikezaki M et al. demonstrated that HSC70 facilitated TGF-β-induced activation of Smad2/3 [15]. In our study, to further elucidate the regulatory effect of HSC70 on Smad3 and thoroughly investigate its relationship with TGF-β1, we used the recombinant protein TGF-β1 to stimulate ESCC cells together with DJ-1 while transfecting cells with HSC70 siRNA. Surprisingly, the results of transwell and 3D tumor spheroid invasion assays indicated that the migration and invasion of K150 and E109 cells were strengthened again after being induced by TGF-β1 (Fig. 6A-D). We then examined the activation of Smad3 in TGF-β1-stimulated ESCC cells. As shown in Fig. 6E-F, the immunofluorescence-stained images vividly demonstrated the re-aggregation of nuclear p-Smad3 in HSPA8-knockdown ESCC cells after stimulated by TGF-β1. Meanwhile, the western blot results also reflected the increased phosphorylation level of Smad3 in ESCC cells after TGF-β1 stimulation (Fig. 6G). These phenomena of Smad3-reactivation indicated that the effect DJ-1/HSC70 acting on Smad3 is separate and concurrent with that of TGF-β1 on Smad3.

### Smad3 transcribes THBS1 to accelerate the metastasis of K150 cells

**Fig. 7:**
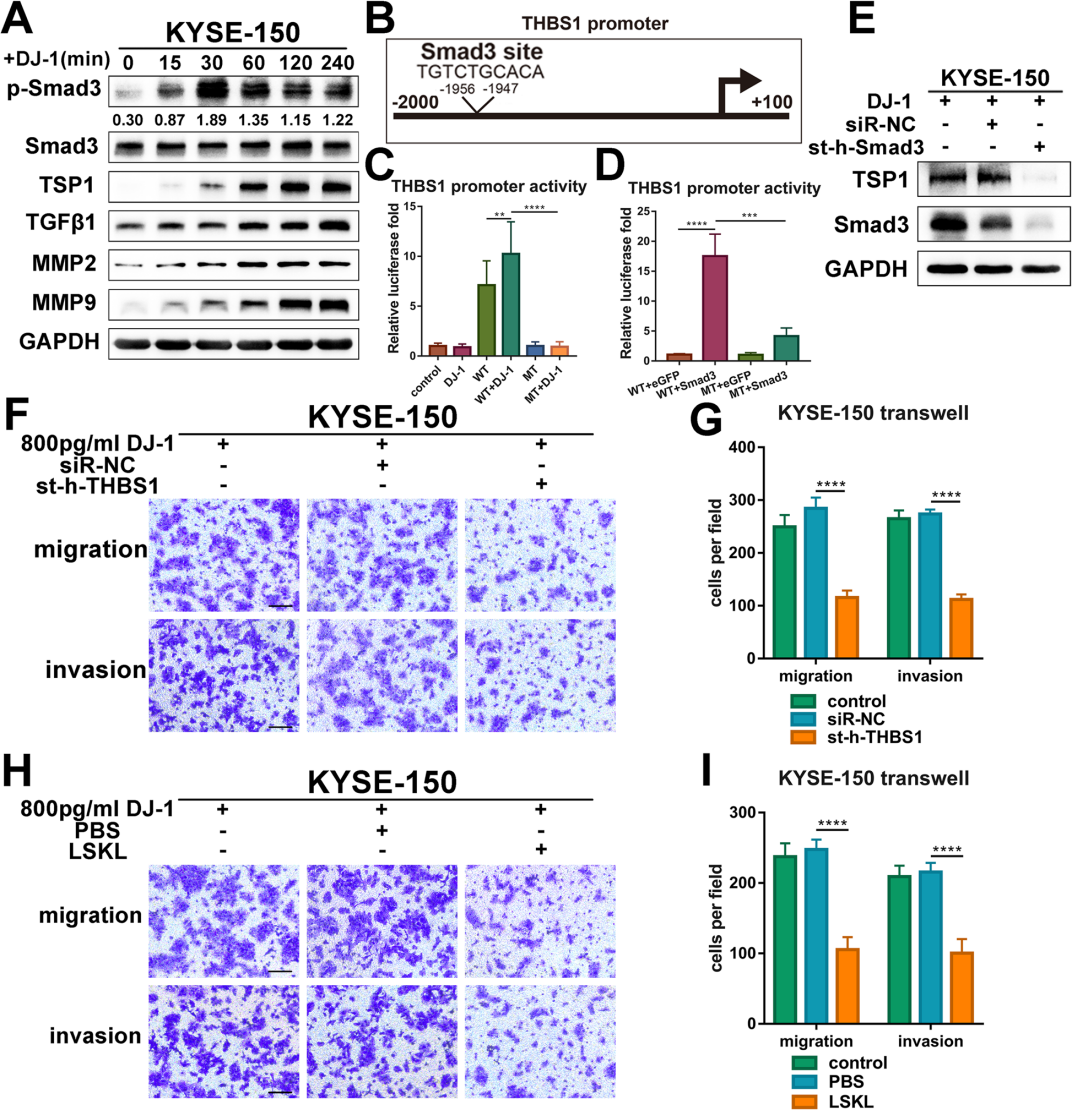
Smad3 transcribes THBS1 to promote the metastasis of K150 cells. **A** Western blot results of prominent proteins correlated with the TGF-β1 signaling pathway in bystander K150 cells at the timing (0, 15, 30, 60, 120 and 240 min) after DJ-1 stimulation. **B** Schematic representation of SMAD3-binding sites on THBS1 promoter. The binding site is located at -1956 ~ -1947 on the THBS1 promoter. **C-D** Luciferase promoter activity was measured by transfecting a PGL3 vector containing the full-length THBS1 promoter (Full) or inserts deleted either in binding site. K150 cells were induced by exogenous DJ-1 (**C**) or transfected with Smad3 overexpression plasmid (**D**). **E** Western blot results of TSP1 and Smad3 in K150 cells transfected with Smad3-knockdown siRNA. **F-G** Representative images and the statistical graph of transwell assays for bystander K150 cells transfected with siRNA targeting THBS1. Scale bars, 100 μm. **H-I** Representative images and the statistical graph of transwell assays for bystander K150 cells measured by LSKL. Scale bars, 100 μm. **p < 0.01, ***p < 0.001, ****p < 0.0001

Considering the restricted regulatory effect of limited exogenous DJ-1, we inferred that there may be a positive feedback loop and DJ-1 probably acts as a catalyst. Smad3 has been widely recognized as an important transcription factor, especially transducing signals from TGF-β superfamily ligands, thus entering the nucleus and activating the transcription of a series of oncogenes [22]. In our study, western blot results suggested that with the passage of time (0–240 min), the ratio of p-Smad3/Smad3 gradually stabilized, and the expression of TSP1, TGF-β1, and MMPs gradually increased in K150 cells (Fig. 7A).

Since phosphorylation and nuclear aggregation of Smad3 are the reasons for DJ-1-induced metastasis, we studied the transcriptional role of Smad3 in K150 cells. Smad3 and DEGs enriched in the TGF-β1 pathway from the RNA-seq results were included in this study. We employed bioinformatics analysis using JASPAR (https://jaspar.genereg.net/) [23] to investigate Smad3 binding sites on the promoter region of above DEGs. We found that there was a Smad3 binding site in the THBS1 promoter region (-1956 ~ -1947), which ranked first in the prediction list (Fig. 7B).

To acquire a deeper understanding of THBS1 transcriptional regulation, we mutated the putative SMAD3-binding sites in the THBS1 promoter and transduced K150 cells with these constructs. Luciferase experiments showed that THBS1 promoter activity was accelerated when stimulated by DJ-1 but inhibited when the SMAD3-binding site was mutated (Fig. 7C). Moreover, we found that transfection with a plasmid overexpressing Smad3 promoted the promoter activity of THBS1 (Fig. 7D). Additionally, western blot analysis revealed that Smad3 silencing by siRNA prominently reduced the expression of THBS1 (Fig. 7E).

After identifying THBS1 as a transcriptional target of Smad3, we investigated its biological function in the metastasis of K150 cells. First, THBS1 siRNA was used to inhibit THBS1 expression in K150 cells. The transfection efficiency is shown in Fig. S7A. Importantly, the migration and invasion stimulated by DJ-1 were decreased in THBS1-knockdown K150 cells compared to control cells in transwell and 3D tumor spheroid invasion assays (Fig. 7F-G and Fig. S7B-C). LSKL, an inhibitor of TSP1, is a latency-associated protein (LAP)-TGFβ-derived tetrapeptide and competitive TGF-β1 antagonist [24]. Therefore, we tested the effect of LSKL on DJ-1-induced metastasis. Transwell and 3D tumor spheroid invasion assays demonstrated that the use of LSKL significantly reduced the migration and invasion induced by DJ-1 in K150 cells (Fig. 7H-I and Fig. S7D-E). In general, these results indicate that after activation by exogenous DJ-1, Smad3 transcribes THBS1 to promote the metastasis of K150 cells.

### Blocking TSP1 weakens the phosphorylation and nucleation of Smad3 and reduces the metastasis of K150 cells in vivo

Thrombospondin 1 (TSP1), encoded by THBS1, has been reported as an activator on latent TGF-β. Previous studies have reported that TSP1 interacts with the LSKL sequence of the N-terminal domain of the LAP of latent TGF-β and induces a conformational change at this site to improve the accessibility of TGF-β to its receptor [25, 26]. In addition, our previous experiments demonstrated that TGF-β1 reactivates Smad3 and enhances the migration and invasion of HSC70-knockdown ESCC cells. Therefore, we speculated that Smad3, TSP1, and TGF-β1 might constitute a positive cycle altogether.

**Fig. 8:**
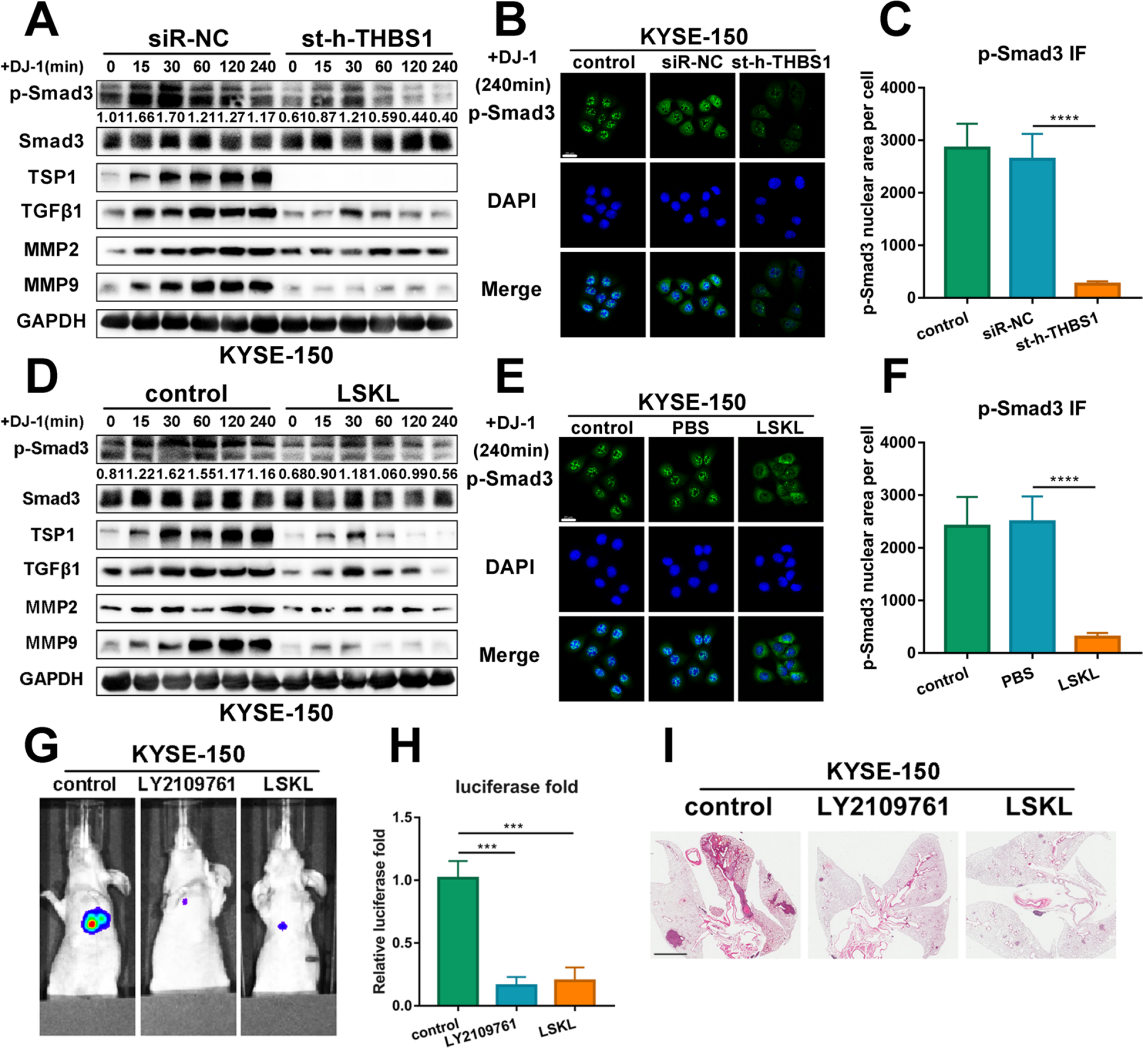
Blocking TSP1 reduces phosphorylation and nuclear aggregation of Smad3 and metastasis in vivo. **A** Western blot results of prominent proteins correlated with the TGF-β1 signaling pathway in bystander K150 cells transfected with siRNA targeting THBS1 at the timing (0, 15, 30, 60, 120 and 240 min) after DJ-1 stimulation. **B-C** Representative IF images of p-Smad3 (green) expression in K150 cells transfected with siRNA targeting THBS1 at the timing (240 min) after DJ-1 stimulation. Scale bar, 20 μm. **D** Western blot results of prominent proteins correlated with the TGF-β1 signaling pathway in bystander K150 cells measured by LSKL at the timing (0, 15, 30, 60, 120 and 240 min) after DJ-1 stimulation. **E–F** Representative IF images of p-Smad3 (green) expression in K150 cells measured by LSKL at the timing (240 min) after DJ-1 stimulation. Scale bar, 20 μm. **G-H** Representative photographs of metastatic nodules were taken from nude mice injected LY2109761 or LSKL by an IVIS imaging system. Quantification of the luciferase was shown. **I** H&E staining was used to characterize the lung metastatic nodules. Scale bar, 2 mm. ***p < 0.001, ****p < 0.0001

To verify our hypothesis, we first transfected siRNA targeting THBS1 into K150 cells. Results of western blotting indicated that compared to the control group, the ratio of p-Smad3/Smad3 in THBS1-knockdown cells suffered a significant decline. At 240 min after DJ-1 stimulation, the p-Smad3/Smad3 ratio was even lower than the initial level. Furthermore, the expression of TGF-β1 and MMPs in the THBS1-knockdown group was markedly decreased compared to that in the control group (Fig. 8A). The translocation of p-Smad3 was examined by immunofluorescence microscopy. As presented in Fig. 8B-C, the level of nuclear aggregation of p-Smad3 greatly decreased in the group that cells transfected with THBS1-knockdown siRNA. Next, we proved that LSKL reduced the phosphorylation and nuclear aggregation of Smad3 using the same methods (Fig. 8D-F). These data revealed that TSP1 responsively activated Smad3 by interacting with TGF-β1, which formed a positive feedback loop.

In addition, to confirm that DJ-1 was a regulator rather than an effector involved in the positive feedback loop, we examined the mRNA levels of PARK7 in ESCC cells by qPCR. The results suggested that exogenous DJ-1 did not strengthen the transcription level of DJ-1 (Fig. S7F).

Having validated the existence of the Smad3/TSP1/ TGF-β1 positive cycle pathway, we predicted that it may be the core effective signaling of DJ-1-induced metastasis and blocking the function of TSP1 or TGF-β1 could interrupt this effect. We established mouse model of pulmonary metastasis to test the therapeutic effects of LY2109761 and LSKL. The results revealed that while DJ-1 was injected through the tail vein equivalently, intraperitoneal injection injected LY2109761 or LSKL greatly suppressed the invasion of K150 cells and alleviated pulmonary metastasis compared with the control group (Fig. 8G-I).

### DJ-1 regulatory pathway proteins are correlated mutually in patient samples and serum DJ-1 has prognostic value in ESCC patients receiving radiotherapy

**Fig. 9:**
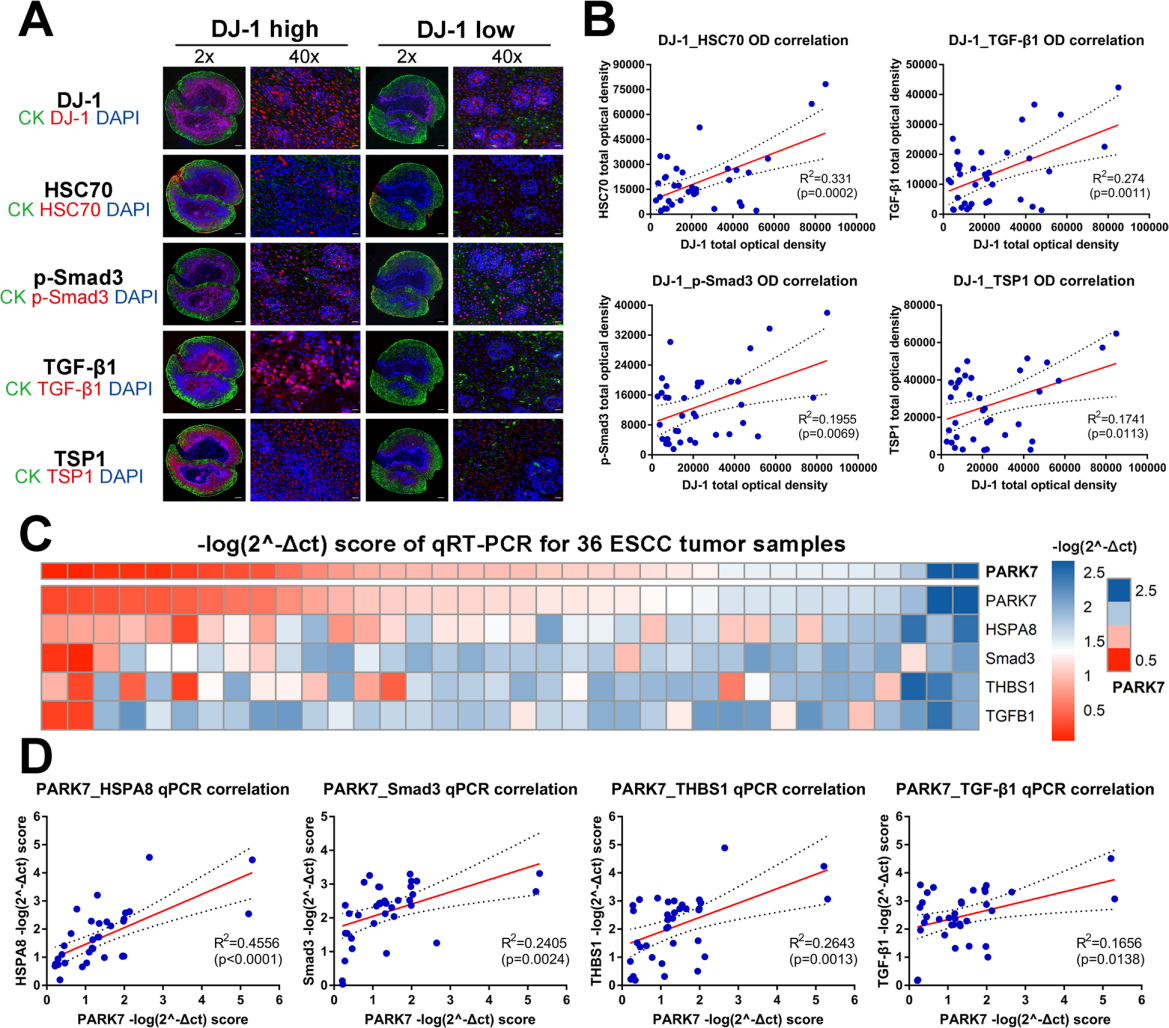
DJ-1 regulatory pathway proteins correlates in patient samples. **A** IF staining for DJ-1, HSC70, p-Smad3, TGF-β1 and TSP1 in ESCC tissues ordered by two groups (DJ-1 high and DJ-1 low). Scale bar, 500 μm (2x) and 20 μm (40x). **B** Correlation analysis of integrated optical density (IOD) between the detected targets with DJ-1. **C** Heatmaps of the score of -log(2^-Δct) from qRT-PCR results of DJ-1, HSC70, p-Smad3, TGF-β1 and TSP1 in ESCC tissues. **D** Correlation analysis of the score of -log(2^-Δct) between the detected targets with DJ-1

To further verify the rationality of the DJ-1 regulatory pathways, we collected 36 ESCC tissues for histological analysis. First, we performed immunohistochemical staining for DJ-1, HSC70, p-Smad3, TSP1 and TGF-β1. We calculated the sum of the optical density per unit area and analyzed the correlation of the integrated optical density (IOD) between the detected targets. The results indicated that there were close correlations between the IOD of DJ-1-HSC70, DJ-1-p-Smad3, DJ-1-TGF-β1, and DJ-1-TSP1 (*R*^*2*^ = 0.281, *p* = 0.0009; *R*^*2*^ = 0.2401, *p* = 0.0024; *R*^*2*^ = 0.1913, *p* = 0.0076; and *R*^*2*^ = 0.2858, *p* = 0.0008, respectively) (Fig. S8A-B). Moreover, immunofluorescence staining for DJ-1, HSC70, p-Smad3, TSP1 and TGF-β1 was performed, and the IOD per area was used as the statistical indicator. Consistent with the IHC results, close correlations of IOD for DJ-1-HSC70, DJ-1-p-Smad3, DJ-1-TGF-β1, and DJ-1-TSP1 (*R*^*2*^ = 0.331, *p* = 0.0002; *R*^*2*^ = 0.1955, *p* = 0.0069; *R*^*2*^ = 0.274, *p* = 0.0011; and *R*^*2*^ = 0.1741, *p* = 0.0113, respectively) were observed (Fig. 9A-B). Additionally, to validate the expression results of histological staining, we used qRT-PCR to determine the expression of the five targets at the mRNA level. The -log(2^-Δct) score was used as a statistical indicator. As presented in Fig. 9C-D, we observed positive correlations for DJ-1-HSC70, DJ-1-p-Smad3, DJ-1-TGF-β1, and DJ-1-TSP1 (*R*^*2*^ = 0.4556, *p* < 0.0001; *R*^*2*^ = 0.2405, *p* = 0.0024; *R*^*2*^ = 0.2643, *p* = 0.0013; and *R*^*2*^ = 0.1656, *p* = 0.0138, respectively). Taken together, these data provide evidence for the existence of DJ-1 regulatory pathways at the human tissue level.

Having determined that DJ-1 induced metastasis in ESCC cells and illustrated its mechanism, we sought to evaluate the predictive value of DJ-1 in survival after radiation in ESCC patients. Follow-up information was collected from 46 patients who had serum samples collected after cumulative 40 Gy dose radiotherapy. Kaplan–Meier survival analysis revealed that high DJ-1 expression in serum after radiotherapy predicted poor overall survival (OS) in patients with ESCC (Fig. S8C, *n* = 46, *p* = 0.0074), while no significant difference was observed in disease free survival (DFS) between the two groups of ESCC patients (Fig. S8D, *n* = 46, *p* = 0.1228). To further evaluate the predictive value of DJ-1 regulatory pathways in the prognosis of patients with ESCC, we calculated the hazard ratios of PARK7, HSPA8, Smad3, THBS1, and TGFB1 in the GSE53625 dataset. However, we did not find any statistically significant differences (Fig. S8E). In summary, although we did not find any value for DJ-1 regulatory pathways in predicting the prognosis of patients with ESCC, serum DJ-1 level could be a useful biomarker to predict survival in patients with ESCC receiving radiotherapy, which still needs to be validated using a large sample of clinical data.

## Discussion

Radiotherapy is an important and effective treatment option for patients with ESCC. However, the existence of RIBE remains a key factor restricting the curative effect of radiotherapy. Recent studies have shown that RIBE plays an important role in promoting metastasis, immune evasion, and enhancing radioresistance in ESCC. For instance, radiation-induced BCLAF1 promotes immunosuppression in ESCC by inhibiting CMTM6 transcription to mediate PD-L1 stabilization [27]. Radiotherapy increases 12-LOX and CCL5 levels in ESCC cells and promotes metastasis [28]. Furthermore, FLT3L secreted by irradiated ESCC cells promotes radiation resistance in paracellular cells [29]. Therefore, future research should explore the mechanism of RIBE and its regulation at the molecular level.

DJ-1 is a 24 kDa small free protein that has the potential for signal transmission between tumor cells. It has been reported that serum DJ-1 is an effective biomarker for the diagnosis and prognosis of pancreatic, colorectal, breast, and endometrial cancer [8, 30–32]. In this study, through the analysis of bioinformatics and clinical samples of ESCC, we found that DJ-1 was highly expressed in tumor tissues and blood samples. High DJ-1 expression was also related to tumor progression and poor prognosis. Interestingly, there was a significant increase in serum DJ-1 levels in patients who received radiation and radiation induced DJ-1 secretion from ESCC cells. These findings suggested that DJ-1 is involved in RIBE in ESCC. Subsequently, a cell co-culture model was constructed to simulate the action of RIBE. Human recombinant protein DJ-1 was used to clarify the function of DJ-1 in RIBE. Our results indicated that radiation-induced secretion of DJ-1 could enhance the metastasis of ESCC cells both in vitro and in vivo.

Moreover, immunofluorescence staining indicated that exogenous DJ-1 was distributed in the cytoplasm and nucleus of ESCC target cells. This suggested that DJ-1 is freely transferred between ESCC cells. Transcriptional co-regulation function is an important role of DJ-1 [33]. For example, under an oxidative condition, DJ-1 binds to and sequesters Keap1 from Nrf2, leading to translocation of Nrf2 into the nucleus to activate various anti-oxidative stress-related genes, thereby decreasing the levels of reactive oxygen species [34]. Afterwards, RNA-seq was performed to investigate the transcriptional changes induced by DJ-1. Feng et al. reported that DJ‑1 activates the Wnt/β‑catenin signaling pathway and mediates the EMT and metastasis in ESCC [11]. Based on our RNA-seq data, we did not get any positive results regarding the Wnt/β‑catenin signaling pathway in the DJ-1-dominated RIBE, but the TGF-β1 pathway was identified by functional enrichment of 434 DEGs. Additionally, blocking the TGF-β1 pathway reduced the effect of DJ-1 on promoting ESCC metastasis.

As a classical tumor-development pathway, TGF-β1 may be activated in many ways. The decrease in tumor progression caused by TGF-β1 blockade was predictable. To explore the regulatory pathway of DJ-1, we identified the protein interacting with DJ-1 by mass spectrometry, then intersected with the DEGs of RNA-seq, and finally screened for the protein HSPA8. A 70 kDa heat-shock cognate protein (HSC70/HSPA8) is a molecular chaperone that plays an essential role in cell survival [35, 36]. HSC70 is a member of the heat-shock protein 70 family of proteins involved in protein folding, translocation or sorting, and regulation of signaling in cells [37]. It has been reported that the complex of HSC70 and MSG1 promotes Smad3-mediated transcription by enhancing coactivators [38]. Furthermore, HSC70 promotes TGF-β-induced Smad2/3 activation in fibroblast NRK-49F cells [15]. In this study, we found that exogenous DJ-1 entered the cytoplasm of ESCC cells and combined with HSC70, promoting the phosphorylation and nuclear aggregation of Smad3, thus enhancing the ESCC metastasis. Except that, we surprisingly found that exogenous TGF-β1 reactivated the Smad3 without the presence of HSC70, which concluded that the effect of DJ-1/HSC70 on Smad3 is separate and concurrent with that of TGF-β1 on Smad3 in ESCC. We consider that the reason for such differences is cellular heterogeneity. Tumor cells have a greater ability of proliferation, survival and migration, and have more approaches in the regulation of the TGF-β1 pathway. For instance, Hsi-Wen Yeh et al. demonstrated that PSPC1 mediates TGF-β1 autocrine signaling and Smad2/3 target switching to promote EMT, stemness and metastasis [39]. Yang Bai et al. reported that FXYD5 regulates the TGF-β/Smad positive feedback loop and drives epithelial‑to‑mesenchymal transition to promote tumor growth and metastasis in ovarian cancer [40]. Therefore, it is necessary to carefully explore the process of TGF-β1 pathway in DJ-1-dominated RIBE.

Because the exogenous DJ-1 used in this study was limited, considering the protein interaction between DJ-1, HSC70 and Smad3, we thought that the activation of Smad3 would be limited. It was unreasonable that the TGF-β1 pathway was activated obviously. Therefore, we predicted that there would be a feedback pathway between TGF-β1 and Smad3. Our subsequent study showed that the entry of p-Smad3 into the nucleus promoted the transcription of THBS1. TSP1(THBS1), also called thrombospondin 1, has a precursor TGF-β1 activation domain in its structure, which specifically binds to the precursor TGF-β1 and converts it into an activated state, thus further initiating the downstream signaling pathway [41]. LSKL is a homologous derivative antagonist of TSP1 that can competitively bind to the precursor TGF-β1 and block its activation [42, 43]. Our research indicated that knocking down THBS1 or using LSKL decreased DJ-1-induced metastasis and weakened the phosphorylation and nuclear aggregation of Smad3. These results suggested that DJ-1 acts as a catalyst in the whole pathway, and the positive feedback pathway of TSP1/TGF-β1/Smad3 is the core effective pathway.

Having explored the pro-metastatic mechanism of action of DJ-1, we aimed to validate the accuracy of this pathway in clinical samples. In our study, 36 immunohistochemical staining, immunofluorescence staining, and qPCR semi-quantitative analysis of esophageal squamous carcinoma tissues indicated that the expression of DJ-1 positively correlated with the expression of HSC70, p-Smad3, TSP1, and TGF-β1, which provided evidence for the existence of DJ-1 regulatory pathways at the human tissue level. Next, we found that DJ-1 levels in serum of patients having received radiotherapy showed encouraging accuracy in predicting prognosis, which suggested the clinical value of DJ-1 as a serum biomarker to predict the efficacy of radiotherapy.

In summary, we have demonstrated that DJ-1 is a master regulator of RIBE in ESCC. First, irradiated ESCC cells secrete DJ-1 into their intercellular compartments. After entering bystander cells, DJ-1 combined with HSC70 promotes p-Smad3 entry into the nucleus, which forms a regulatory pathway. Next, the transcriptional activity of THBS1 is elevated, and the expressed TSP1 bind to the precursor TGF-β1 to promote Smad3 phosphorylation and nuclear aggregation, which formed a positive feedback pathway. Finally, the metastasis of bystander ESCC cells is increased. Clinical specimens of ESCC demonstrated the value of serum DJ-1 in predicting the radiotherapy response. Our findings not only reveal the mechanism by which DJ-1 regulates metastasis under irradiation but also provide a potential new clinical biomarker to predict the efficacy of radiotherapy and new therapeutic targets to overcome the RIBE of ESCC.

## Conclusions

Irradiation induces DJ-1 secretion in ESCC cells. Secreted DJ-1 enhances the metastasis of ESCC cells both in vitro and in vivo. Mechanistically, exogenous DJ-1 enters bystander cells to initiate the activation of the TGF-β1 pathway via the DJ-1/HSC70/Smad3 signaling axis. The TSP1/TGF-β1/Smad3 positive feedback pathway constitutes the core pathway that promotes ESCC metastasis. This study provides a useful biomarker for predicting the efficacy of radiotherapy and a potential therapeutic target for reversing RIBE.

## Supplementary Information

**Additional file 1: Figure S1.** Pattern diagram of co-culture model in vitro experiments.

**Additional file 2: Figure S2.** DJ-1 expression is examined in public database. A. DJ-1 expression in normal, EA and ESCC tissues were assessed using data from the TCGA database. B. DJ-1 expression in ESCC tissues at T stage from the TCGA database. C. DJ-1 expression in normal and ESCC tissues were assessed using data from the GSE53625 data set. D. Kaplan-Meier survival analysis of overall survival of ESCC patients with different histological DJ-1 levels from the GSE53625 data set. *p<0.05, **p<0.01.

**Additional file 3: Figure S3.** The detail data of DJ-1 secretion from irradiated cells. A-B. ELISA results of DJ-1 expression in medium of ESCC cells at multiple timing (0.5, 1, 2, 4, 8, 12 and 24h) after receiving multiple doses (0, 2, 4, 6, 8 and 10Gy) of irradiation. C-F. Western blot results and statistical graphs of intracellular DJ-1 levels at multiple timing (0.5, 1, 2, 4, 8, 12 and 24h) after receiving 8Gy irradiation. *p<0.05, **p<0.01, ***p<0.001, ****p<0.0001.

**Additional file 4: Figure S4.** The DJ-1 intracellular localization and supplemental data of activating TGF-β1 pathway. A. Fluorescence co-localization analysis of His-tag and DJ-1 in ESCC bystander cells. B. The volcano map of DEGs from RNAseq analysis. C-D. Representative IF images and the statistical graph of 3D tumor spheroid invasion assays for bystander ESCC cells (green) measured by LY2109761. Scale bars, 200μm. **p<0.01, ***p<0.001.

**Additional file 5: Figure S5.** The supplemental data of DJ-1 interacts with HSC70 in promoting bystander ESCC cells metastasis. A. Photograph of the gel with samples of DJ-1 co-immunoprecipitation treated by Coomassie brilliant blue staining. B. Western blot results of HSC70 in K150 and E109 cells transfected with HSC70-knockdown siRNA. C-D. Fluorescence co-localization analysis of DJ-1 and HSC70 in K150 and E109 cells. E-F. Representative IF images and the statistical graph of 3D tumor spheroid invasion assays for bystander K150 and E109 cells (green) transfected with siRNA targeting HSC70. Scale bars, 200μm. **p<0.01.

**Additional file 6: Figure S6.** The supplemental data of effects DJ-1/HSC70 conducts on Smad3. A-B. The statistical graph of fluorescence intensity of p-Smad3 (green) and HSC70 (red) in IF images from fig5B results. C. Western blot results of Smad3 in K150 and E109 cells transfected with Smad3-knockdown siRNA. D-E. Representative IF images and the statistical graph of 3D tumor spheroid invasion assays for bystander K150 and E109 cells (green) transfected with siRNA targeting Smad3. Scale bars, 200μm. *p<0.05, **p<0.01, ***p<0.001.

**Additional file 7: Figure S7.** The supplemental data of TSP1 functions in DJ-1-induced metastasis. A. Western blot results of TSP1 in K150 cells transfected with THBS1-knockdown siRNA. B-C. Representative IF images and the statistical graph of 3D tumor spheroid invasion assays for bystander K150 cells (green) transfected with siRNA targeting THBS1. Scale bars, 200μm. D-E. Representative IF images and the statistical graph of 3D tumor spheroid invasion assays for bystander K150 cells (green) measured by LSKL. Scale bars, 200μm. F. qPCR results of PARK7 mRNA expression in bystander ESCC cells stimulated by exogenous DJ-1. *p<0.05.

**Additional file 8: Figure S8.** IHC staining results of DJ-1 regulatory pathway proteins in patient samples and prognostic analysis of DJ-1. A. IHC staining for DJ-1, HSC70, p-Smad3, TGF-β1 and TSP1 in ESCC tissues ordered by two groups (DJ-1 high and DJ-1 low). Scale bar, 100μm (10x) and 50μm (40x). B. Correlation analysis of integrated optical density (IOD) between the detected targets with DJ-1. C-D. Kaplan-Meier survival analysis of overall survival (OS) and disease-free survival (DFS) of ESCC patients who collected serum samples after cumulative 40Gy dose radiotherapy. E. Forest plot of hazard ratios of PARK7, HSPA8, Smad3, THBS1 and TGFB1 in the GSE53625 data set.

**Additional file 9: Table S1.** Clinical data for 177 ESCC serum samples used for ELISA.

**Additional file 10: Table S2.** Clinical data for 46 serum samples of ESCC patients receiving similar treatments, used for ELISA and survival analysis.

**Additional file 11: Table S3.** Clinicopathological data for 36 ESCC tissues and non-neoplastic ESCC tissue samples used for histological detections.

**Additional file 12: Table S4.** List of antibodies for western blotting, immunofluorescence, immunohistochemistry or Co-immunoprecipitation analyses.

**Additional file 13: Table S5.** Probes for siRNAs and plasmid/primers for the luciferase reporter vectors and primers for RT-qPCR.

**Additional file 14: Table S6.** The whole transcriptome results detected by RNA-seq.

**Additional file 15: Table S7.** The total proteins identified from the LC–MS/MS.

## Data Availability

The datasets used and/or analyzed during the current study are available from the corresponding author upon reasonable request.

## Abbreviations

DJ-1/PARK7: Parkinson disease protein 7
TGF-β1: Transforming growth factor beta-1 proprotein
RIBE: Radiation-induced bystander effect
ESCC: Esophageal squamous cell carcinoma
HSC70/HSPA8: Heat shock cognate 71 kDa protein
Smad3: Mothers against decapentaplegic homolog 3
TSP1/THBS1: Thrombospondin-1
H-RAS: Harvey rat sarcoma viral oncogene homolog
TCGA: The cancer genome atlas
GEO: Gene Expression Omnibus
MMP2: Matrix metallopeptidase 2
MMP9: Matrix metallopeptidase 9
KEGG: Kyoto Encyclopedia of Genes and Genomes
HE: Haematoxylin and eosin
IOD: Integrated optical density
OS: Overall survival
DFS: Disease free survival
BCLAF1: Bcl-2-associated transcription factor 1
CMTM6: CKLF-like MARVEL transmembrane domain-containing protein 6
PD-L1: Programmed cell death 1 ligand 1
12-LOX: Dioxygenase 12-lipoxygenase
CCL5: Chemokine (C–C motif) ligand 5
FLT3L: FMS-related tyrosine kinase 3 ligand
Nrf2: Nuclear factor erythroid 2-related factor 2
