# Supplementary figures and images for: Irradiation induces DJ-1 secretion from esophageal squamous cell carcinoma cells to accelerate metastasis of bystander cells via a TGF-β1 positive feedback loop
Source: J Exp Clin Cancer Res. 2022 Aug 26;41:259. doi: 10.1186/s13046-022-02471-6 (PMC9413943; doi:10.1186/s13046-022-02471-6)

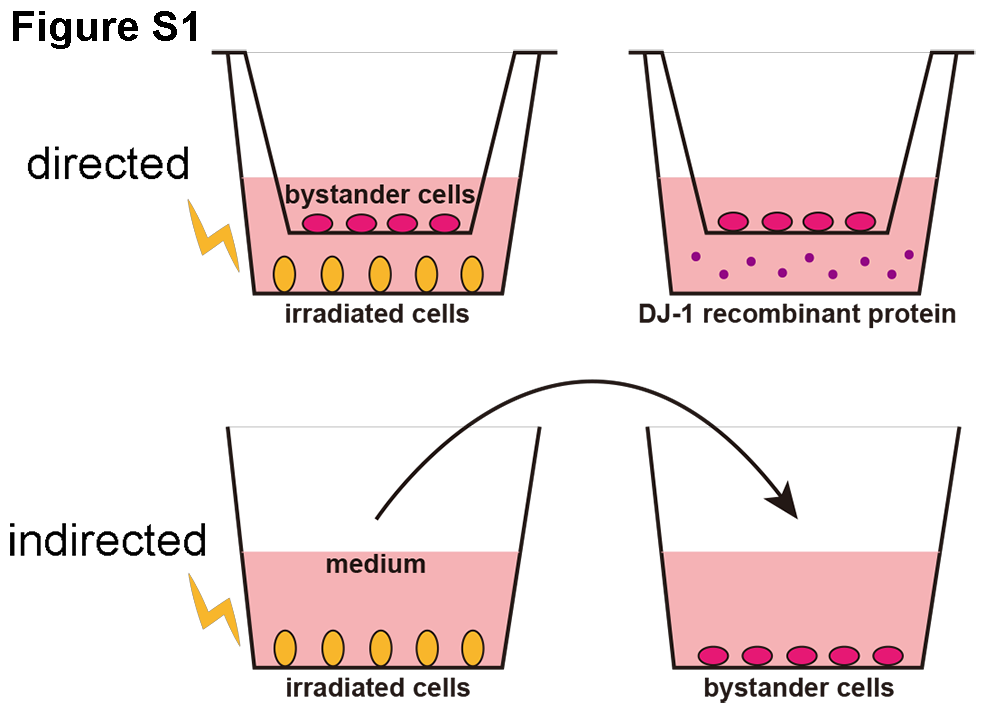

Supplement: Supplementary file 1 — Additional file 1: Figure S1. Pattern diagram of co-culture model in vitro experiments. [file 13046_2022_2471_MOESM1_ESM.tif]

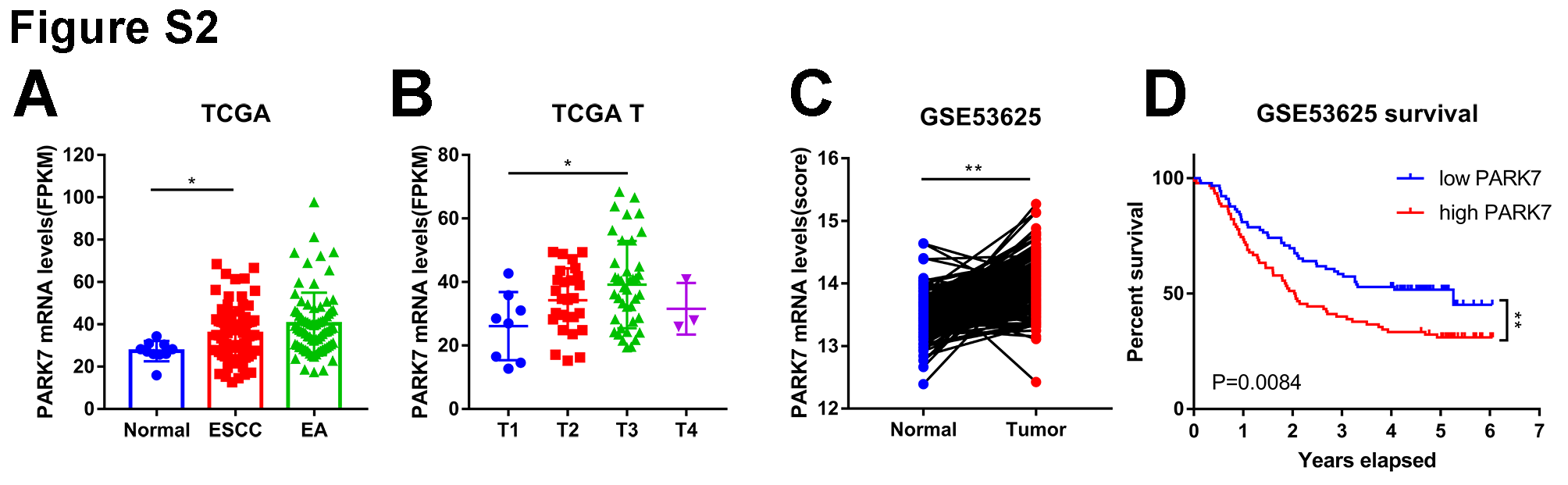

Supplement: Supplementary file 2 — Additional file 2: Figure S2. DJ-1 expression is examined in public database. A. DJ-1 expression in normal, EA and ESCC tissues were assessed using data from the TCGA database. B. DJ-1 expression in ESCC tissues at T stage from the TCGA database. C. DJ-1 expression in normal and ESCC tissues were assessed using data from the GSE53625 data set. D. Kaplan-Meier survival analysis of overall survival of ESCC patients with different histological DJ-1 levels from the GSE53625 data set. *p<0.05, **p<0.01. [file 13046_2022_2471_MOESM2_ESM.tif]

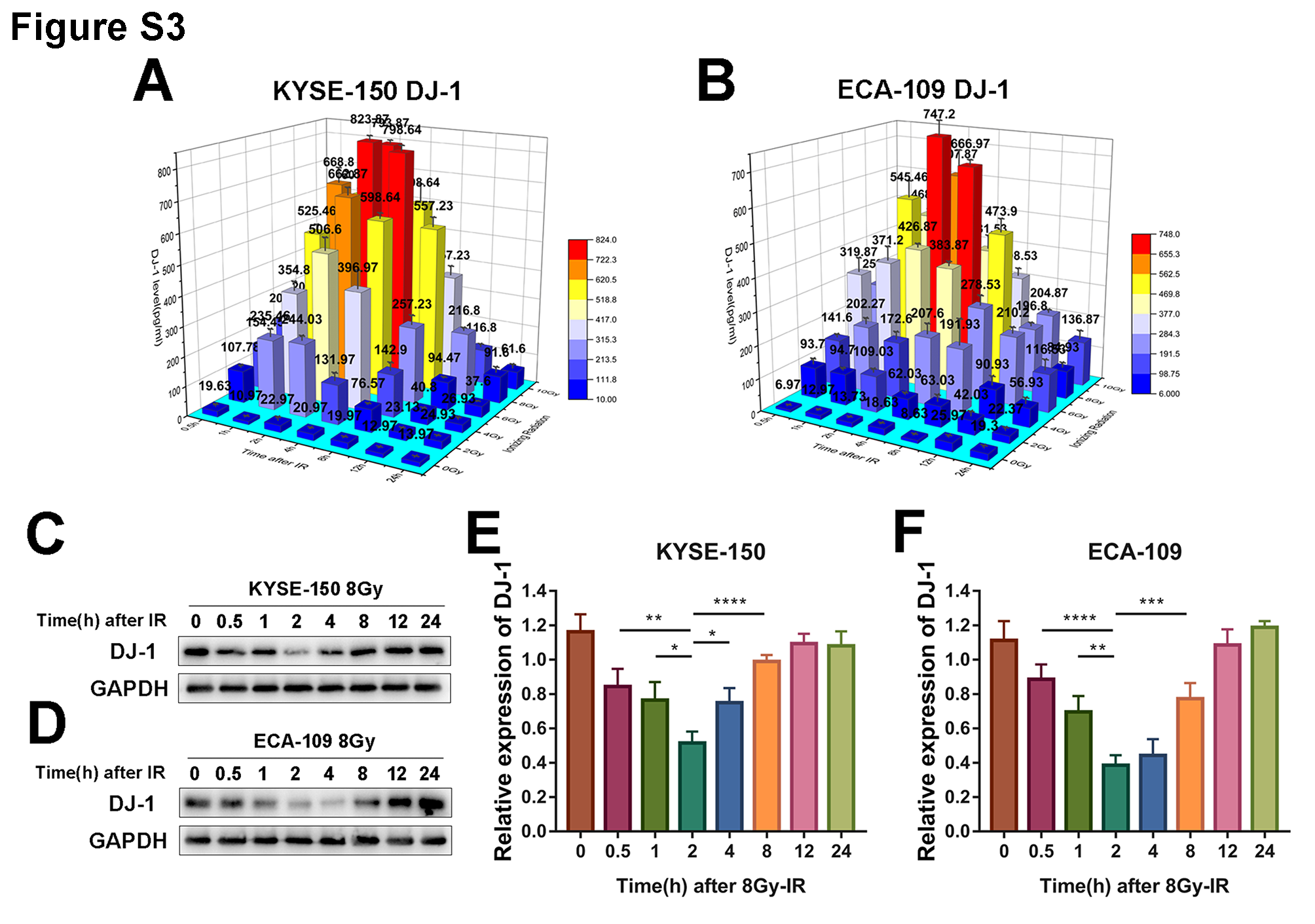

Supplement: Supplementary file 3 — Additional file 3: Figure S3. The detail data of DJ-1 secretion from irradiated cells. A-B. ELISA results of DJ-1 expression in medium of ESCC cells at multiple timing (0.5, 1, 2, 4, 8, 12 and 24h) after receiving multiple doses (0, 2, 4, 6, 8 and 10Gy) of irradiation. C-F. Western blot results and statistical graphs of intracellular DJ-1 levels at multiple timing (0.5, 1, 2, 4, 8, 12 and 24h) after receiving 8Gy irradiation. *p<0.05, **p<0.01, ***p<0.001, ****p<0.0001. [file 13046_2022_2471_MOESM3_ESM.tif]

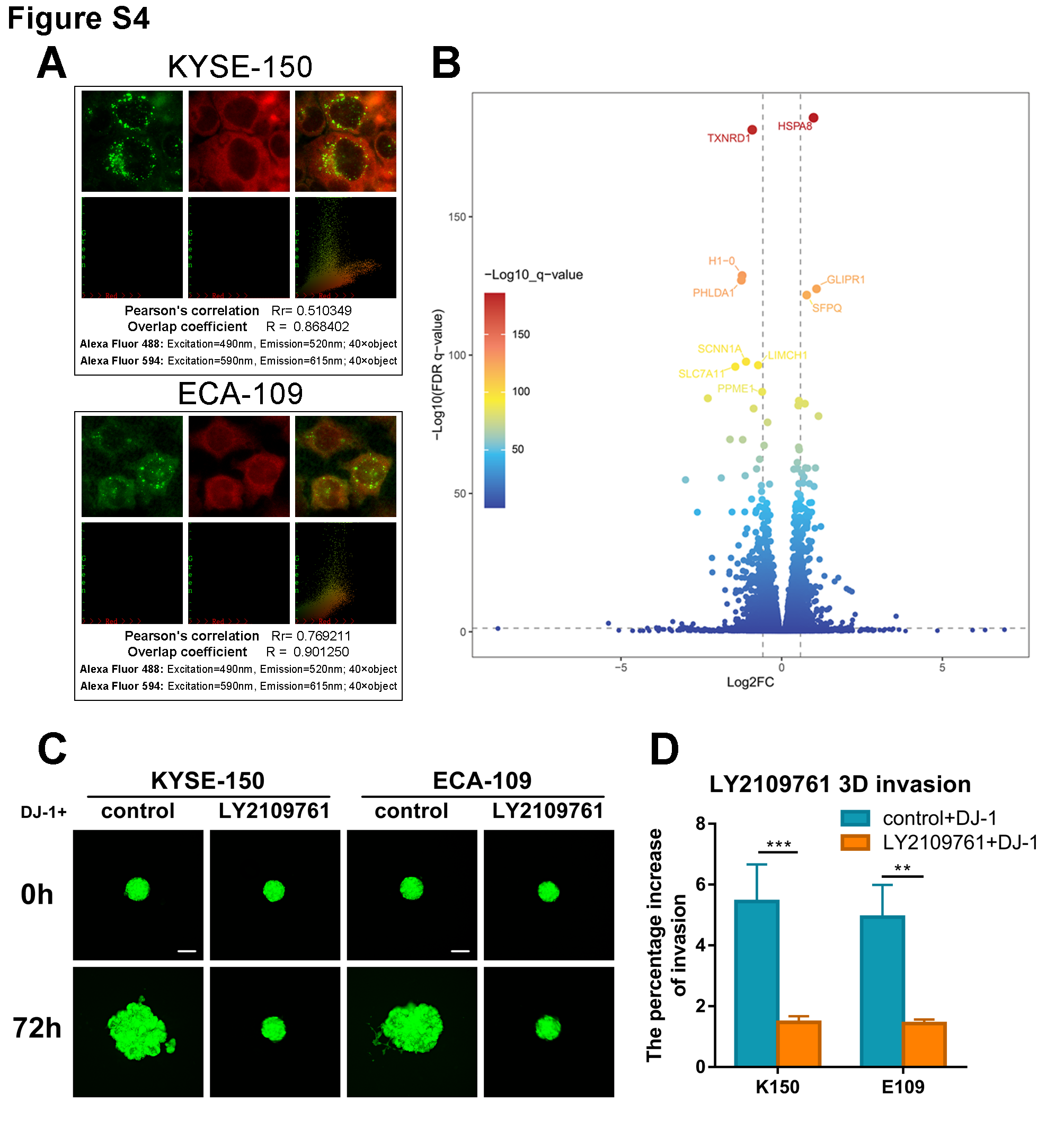

Supplement: Supplementary file 4 — Additional file 4: Figure S4. The DJ-1 intracellular localization and supplemental data of activating TGF-β1 pathway. A. Fluorescence co-localization analysis of His-tag and DJ-1 in ESCC bystander cells. B. The volcano map of DEGs from RNAseq analysis. C-D. Representative IF images and the statistical graph of 3D tumor spheroid invasion assays for bystander ESCC cells (green) measured by LY2109761. Scale bars, 200μm. **p<0.01, ***p<0.001. [file 13046_2022_2471_MOESM4_ESM.tif]

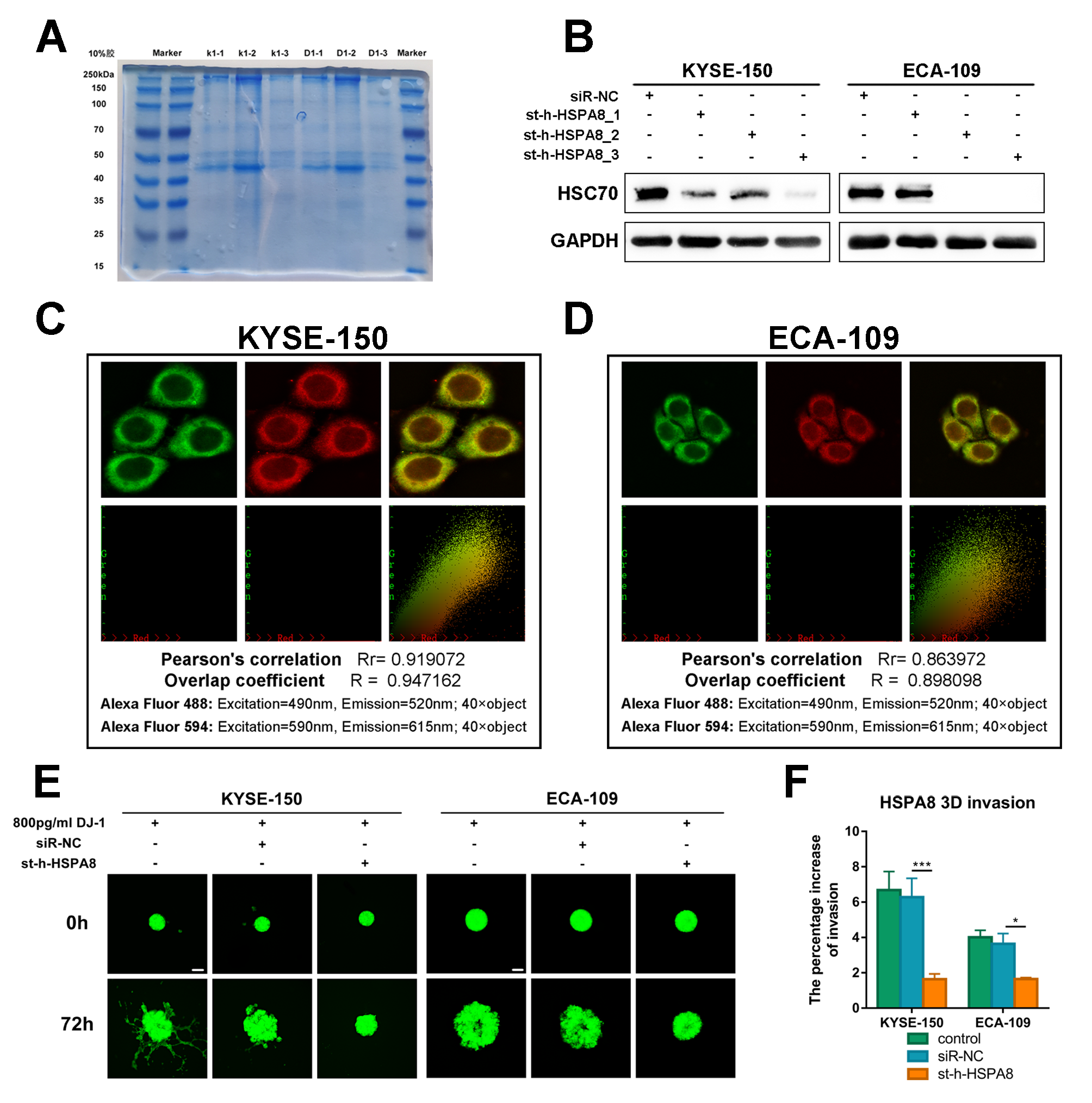

Supplement: Supplementary file 5 — Additional file 5: Figure S5. The supplemental data of DJ-1 interacts with HSC70 in promoting bystander ESCC cells metastasis. A. Photograph of the gel with samples of DJ-1 co-immunoprecipitation treated by Coomassie brilliant blue staining. B. Western blot results of HSC70 in K150 and E109 cells transfected with HSC70-knockdown siRNA. C-D. Fluorescence co-localization analysis of DJ-1 and HSC70 in K150 and E109 cells. E-F. Representative IF images and the statistical graph of 3D tumor spheroid invasion assays for bystander K150 and E109 cells (green) transfected with siRNA targeting HSC70. Scale bars, 200μm. **p<0.01. [file 13046_2022_2471_MOESM5_ESM.tif]

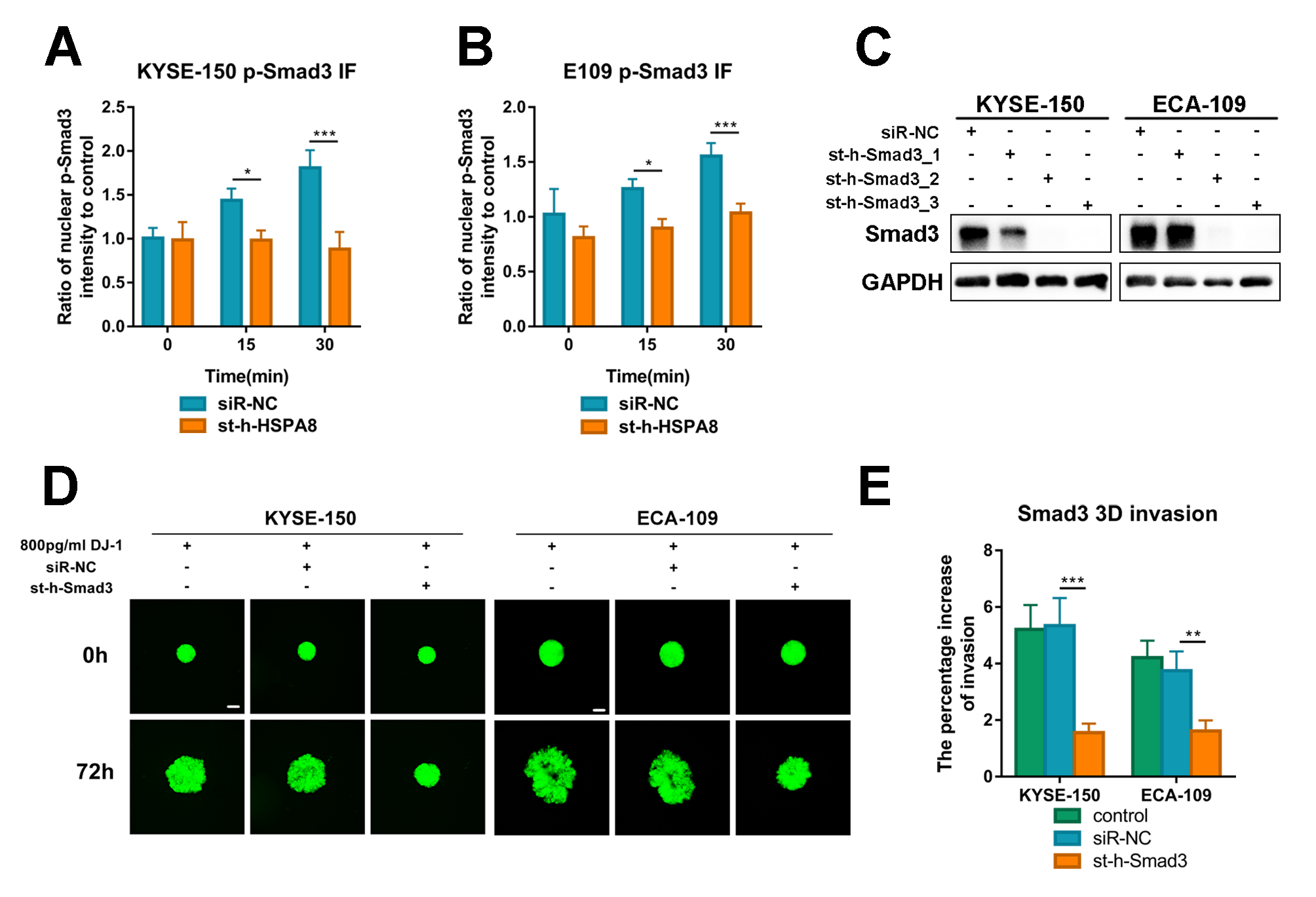

Supplement: Supplementary file 6 — Additional file 6: Figure S6. The supplemental data of effects DJ-1/HSC70 conducts on Smad3. A-B. The statistical graph of fluorescence intensity of p-Smad3 (green) and HSC70 (red) in IF images from fig5B results. C. Western blot results of Smad3 in K150 and E109 cells transfected with Smad3-knockdown siRNA. D-E. Representative IF images and the statistical graph of 3D tumor spheroid invasion assays for bystander K150 and E109 cells (green) transfected with siRNA targeting Smad3. Scale bars, 200μm. *p<0.05, **p<0.01, ***p<0.001. [file 13046_2022_2471_MOESM6_ESM.tif]

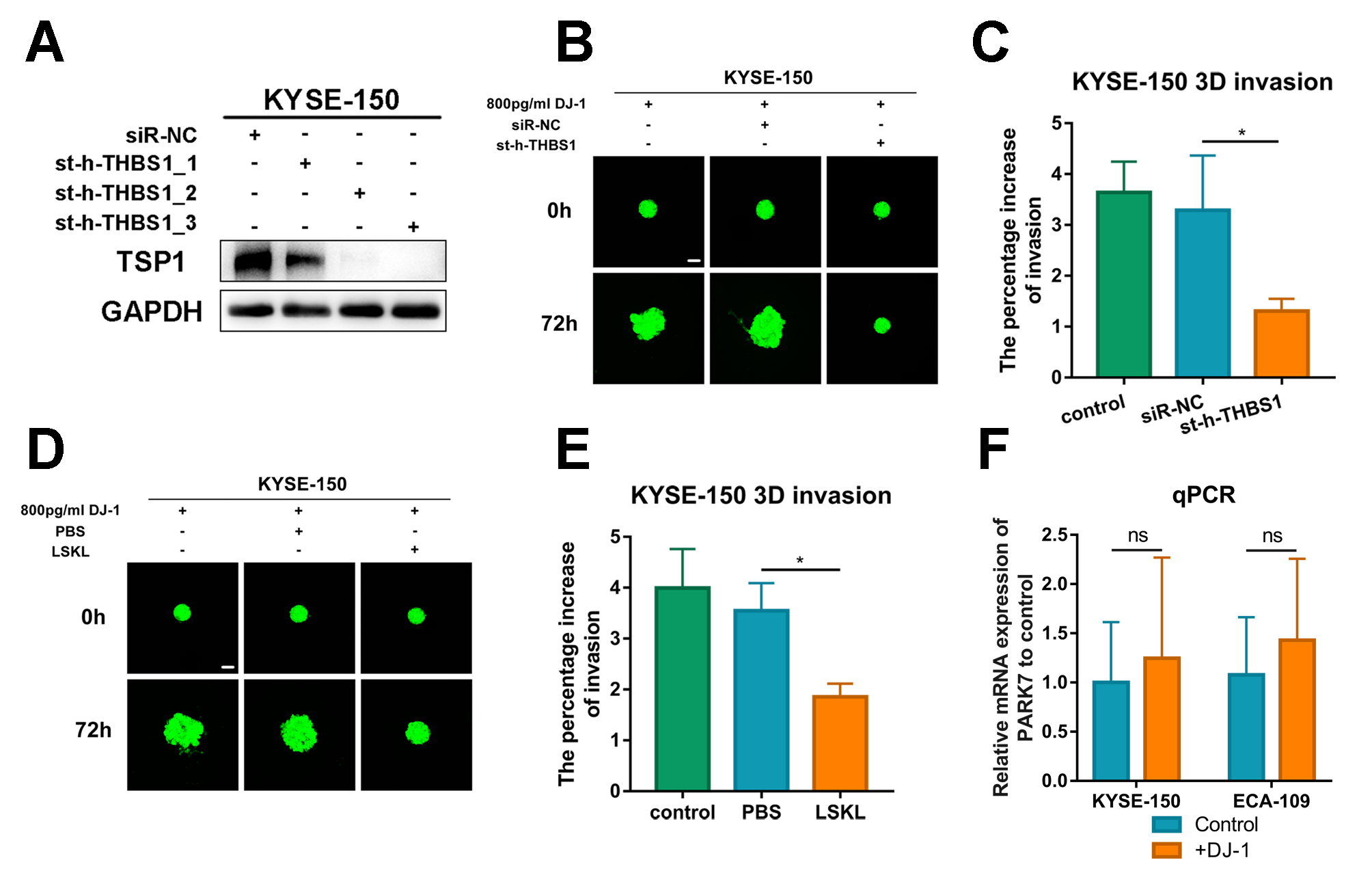

Supplement: Supplementary file 7 — Additional file 7: Figure S7. The supplemental data of TSP1 functions in DJ-1-induced metastasis. A. Western blot results of TSP1 in K150 cells transfected with THBS1-knockdown siRNA. B-C. Representative IF images and the statistical graph of 3D tumor spheroid invasion assays for bystander K150 cells (green) transfected with siRNA targeting THBS1. Scale bars, 200μm. D-E. Representative IF images and the statistical graph of 3D tumor spheroid invasion assays for bystander K150 cells (green) measured by LSKL. Scale bars, 200μm. F. qPCR results of PARK7 mRNA expression in bystander ESCC cells stimulated by exogenous DJ-1. *p<0.05. [file 13046_2022_2471_MOESM7_ESM.tif]

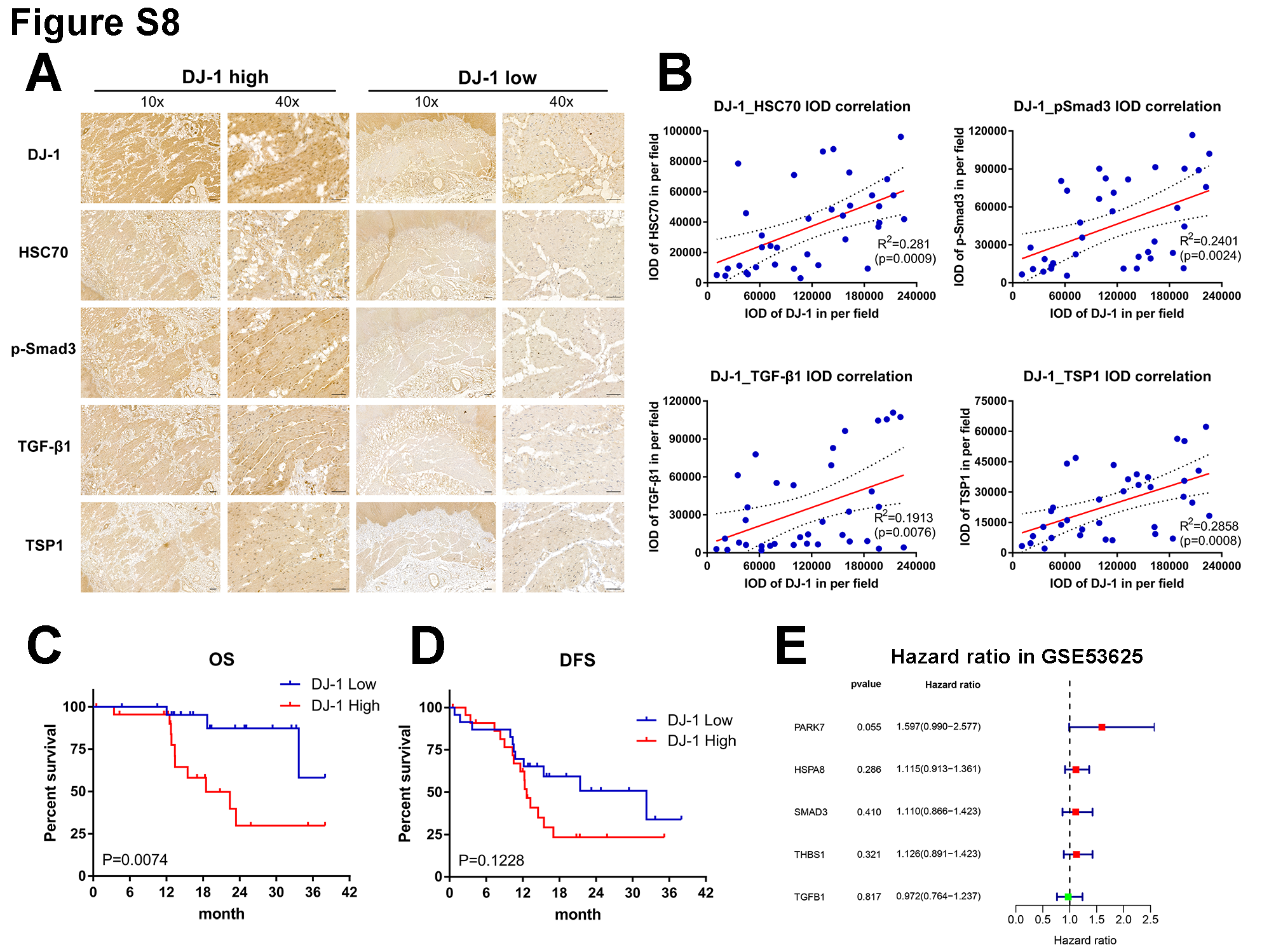

Supplement: Supplementary file 8 — Additional file 8: Figure S8. IHC staining results of DJ-1 regulatory pathway proteins in patient samples and prognostic analysis of DJ-1. A. IHC staining for DJ-1, HSC70, p-Smad3, TGF-β1 and TSP1 in ESCC tissues ordered by two groups (DJ-1 high and DJ-1 low). Scale bar, 100μm (10x) and 50μm (40x). B. Correlation analysis of integrated optical density (IOD) between the detected targets with DJ-1. C-D. Kaplan-Meier survival analysis of overall survival (OS) and disease-free survival (DFS) of ESCC patients who collected serum samples after cumulative 40Gy dose radiotherapy. E. Forest plot of hazard ratios of PARK7, HSPA8, Smad3, THBS1 and TGFB1 in the GSE53625 data set. [file 13046_2022_2471_MOESM8_ESM.tif]
